# Long lasting insecticidal bed nets ownership, access and use in a high malaria transmission setting before and after a mass distribution campaign in Uganda

**DOI:** 10.1371/journal.pone.0191191

**Published:** 2018-01-18

**Authors:** Humphrey Wanzira, Thomas Eganyu, Ronald Mulebeke, Fred Bukenya, Dorothy Echodu, Yeka Adoke

**Affiliations:** 1 Pilgrim Africa, Kampala, Uganda; 2 Makerere University School of Public Health, Kampala, Uganda; Centro de Pesquisas Rene Rachou, BRAZIL

## Abstract

**Introduction:**

Uganda is conducting a second mass LLIN distribution campaign and Katakwi district recently received LLINs as part of this activity. This study was conducted to measure the success of the campaign in this setting, an area of high transmission, with the objectives to estimate LLIN ownership, access and use pre and post campaign implementation.

**Methods:**

Two identical cross sectional surveys, based on the Malaria Indicator Survey methodology, were conducted in three sub-counties in this district (Kapujan, Magoro and Toroma), six months apart, one before and another after the mass distribution campaign. Data on three main LLIN indicators including; household LLIN ownership, population with access to an LLIN and use were collected using a household and a women’s questionnaire identical to the Malaria Indicator Survey.

**Results:**

A total of 601 and 607 households were randomly selected in survey one and two respectively. At baseline, 60.57% (56.53–64.50) of households owned at least one net for every two persons who stayed in the household the night before the survey which significantly increased to 70.35% (66.54–73.96) after the campaign (p = 0.001). Similarly, the percentage of the household population with access to an LLIN significantly increased from 84.76% (82.99–86.52) to 91.57% (90.33–92.81), p = 0.001 and the percentage of household population that slept under an LLIN the night before the survey also significantly increased from 56.85% (55.06–58.82) to 81.72% (76.75–83.21), p = 0.001.

**Conclusion:**

The LLIN mass campaign successfully achieved the national target of over eighty-five percent of the population with access to an LLIN in this setting, however, universal household coverage and use were fourteen and three percent points less than the national target respectively. This is useful for malaria programs to consider during the planning of future campaigns by tailoring efforts around deficient areas like mechanisms to increase universal coverage and behavior change communication.

## Introduction

Long-lasting insecticidal nets (LLINs) are core malaria prevention tool that is recommended by the World Health Organization (WHO) for use by people at risk of contracting malaria especially in malaria endemic countries [1]. This is because LLINs have been shown to reduce malaria incidence among children under five years and pregnant women by up to 50 percent and all-cause mortality in children by about 20 percent [2–4]. Additionally, this is the most cost-effective malaria prevention intervention in regions of high malaria transmission intensity [4–5] and therefore increasing LLIN coverage and use is the most promoted malaria vector control prevention strategy in malaria endemic countries such as Uganda [1,6–7].

In 2009, Uganda adopted the World Health Organization (WHO) recommended LLIN universal coverage (defined as one LLIN for every two persons) [5,7]. There have been several approaches the country has used to rapidly achieve universal LLIN coverage however, the most cost-effective way has been through mass distribution campaigns, aiming for high and equitable coverage [4–5]. The first mass LLIN distribution campaign was conducted in 2013, where over 20 million nets were distributed to over 41 million individuals [7–9] and the second mass campaign of 2017 is nearing completion, with over 24 million LLINs estimated to be distributed [10]. The overall aim of the campaigns is to ensure that at least 85 percent of the targeted population has access to an LLIN and to achieve 85 percent utilization of the distributed bed nets [7].

So as to measure the effectiveness of the 2013 mass distribution campaign exercise against the set targets for LLIN access and use, Uganda conducted its second Malaria Indicator Survey in 2014 (2014 MIS) which showed that 62 percent of households surveyed achieved universal coverage, 79 percent of surveyed population had access to an LLIN and only 69 percent used it the night before [11]. However, it was not apparent as to the extent that the mass campaigns contributed to these estimates because of a lack of definitive baseline, especially considering that there have always been other channels from which the community population receive LLINs including the routine antenatal clinics and private health care facilities [7]. The alternative quoted baseline at the time was from the 2009 MIS [12] that had been conducted four years prior to the 2013 mass distribution campaign, estimates that were not accurate because they had been over taken by events during that long time interval.

Therefore, in an effort to derive an accurate measure of effectiveness of the LLIN mass campaign to achieve the set LLIN targets, we conducted two surveys, one three months before the mass distribution and the other three months after the mass distribution campaign. Such accurate estimates are essential for the National Malaria Control Program (NMCP) especially during the planning of future mass campaigns so as to focus implementation on priority deficient areas at baseline and maximize the campaigns’ potential for success. Therefore, the overall aim of this study was to estimate LLIN ownership, access and use before and after the mass distribution campaign among households in a high malaria transmission setting.

## Methods

### Study design and setting

This study utilizes two methodologically identical cross-sectional surveys conducted in three sub-counties in Katakwi district (Kapujan, Toroma and Magoro), a setting of high malaria transmission intensity, located in the eastern region of Uganda. According to the 2016 Health Management and Information System (HMIS) malaria data, the three sub-counties reported a malaria burden of 4,037 cases, translating into a positivity rate of 30% [13]. In 2013, the three sub-counties received 28,764 LLINs for a population of 53,477 in the first mass distribution campaign and in the 2017, they received 36,314 LLINs for a population of 36,314 during the most recent campaign (one net for every two persons).

This study is part of a larger quasi experimental project assessing novel ways of malaria prevention, with Kapujan residents selected to receive mass drug administration (MDA) with dihydroartemesinin-piperaquine and indoor residual spraying (IRS), Toroma residents receiving IRS only while Magoro is the control sub-county.

The two surveys were conducted six months apart, the first in November to December 2016 while the second was in June to July 2017. In March 2017, in-between these two surveys, the three sub-counties received the free LLINs under the second national mass distribution campaign, and the details of the distribution approach are contained in the 2016 National LLIN implementation guidelines [10].

### Sample size and sampling of households

The unit of selection was the household and the sample size for this survey was based on the assumptions of the main project, that a sample size of 300 children per survey (200 households x 1.5 children per household) aged 6–14 years would have a power of 80% to reject the alternative hypothesis that there is a difference in estimates of Parasite Rate (PR) assuming that the absolute difference in PR is no greater than 10% across a range of PRs in the community surveys of 20–80%.

**Fig 1:**
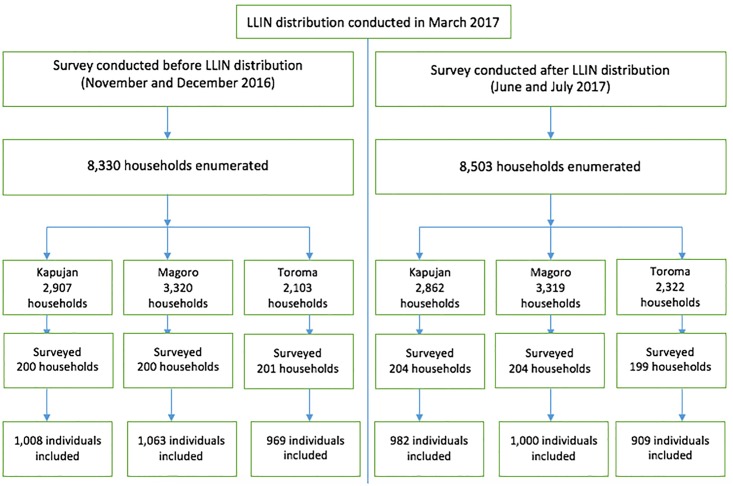
Study profile.

**Fig 2:**
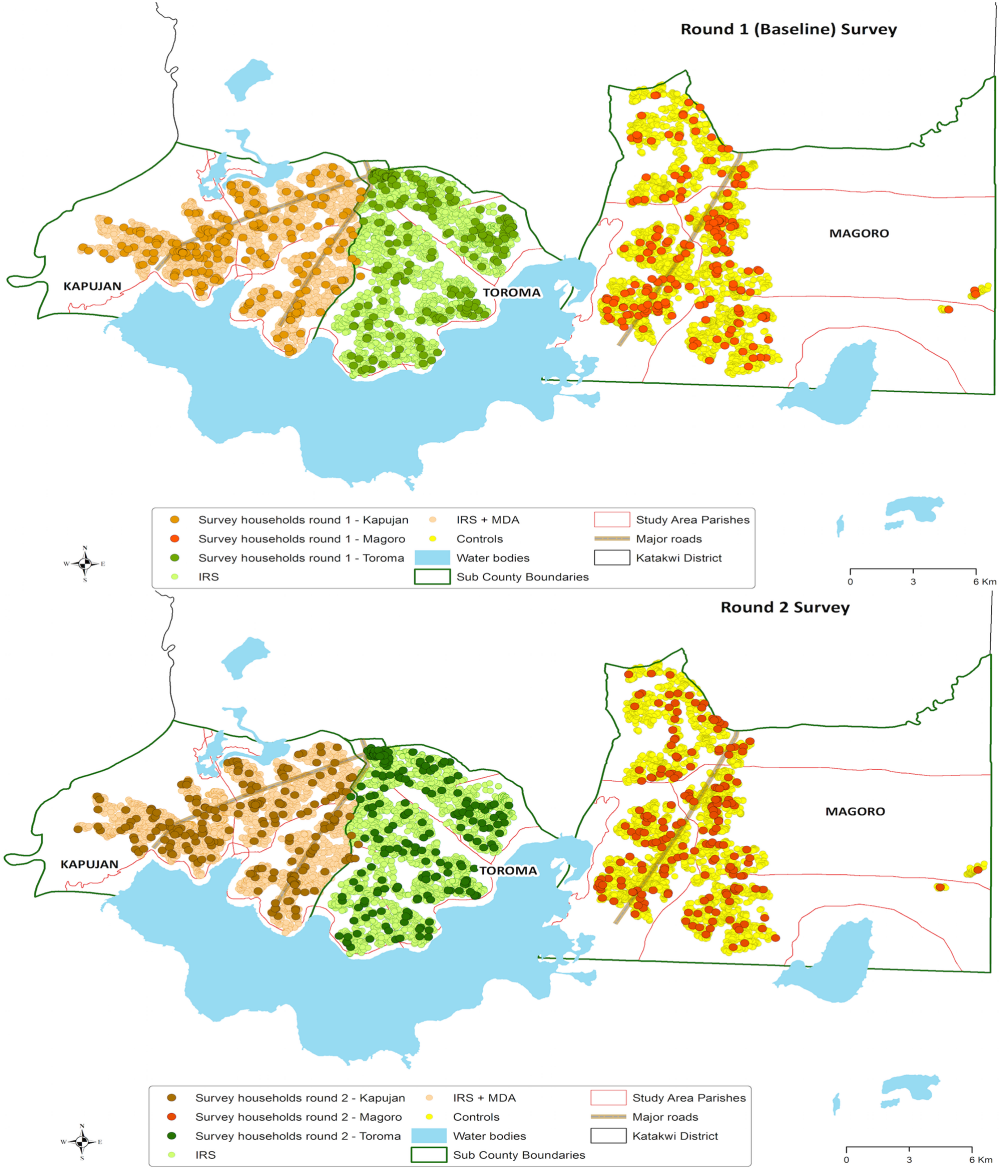
Map showing the distribution of households surveyed in each sub-county.

In order to create a non-biased sampling frame, all households in the three sub-counties were enumerated before each survey. Thereafter a computerized number generator using a probability proportion to village size approach was used to randomly select approximately 200 households from each of the sub-counties (Figs 1 and 2).

### Study questionnaires and variables

Two questionnaires were used in the surveys: a household questionnaire and a woman’s questionnaire for all women aged 15–49 years in selected households. Both instruments were based on of the Malaria Indicator Survey questionnaires developed by the Roll Back Malaria Partnership Monitoring and Evaluation Reference Group [11]. In each household, an adult household member aged ≥18 years, if possible the head of household, was asked to respond to the household questionnaire and all women aged 15–49 in selected households were asked to participate in the woman’s questionnaire. Written consent was sought from the head of households or their designate to participate in the surveys and all interviewed women.

The household questionnaire was used to list all usual members and visitors in the selected households and also inquire about bed net ownership, access and use in addition to other information like household characteristics and assets. The woman’s questionnaire was used to collect information from all women aged 15–49 years on background characteristics including age, recent reproductive history including current pregnancy and preventive malaria approaches like the use of bed nets.

### Study outcomes

The three main LLIN outcome variables are; 1) percentage of households with at least one net for every two persons who stayed in the household the night before; 2) percentage with access to an LLIN (population that could sleep under an LLIN if each LLIN in the household were used by up to two people) and percentage of the de-facto household population that slept under an LLIN the last night. De-facto household members are all people present in the household on night of the survey including visitors. Other outcome variables considered included: percentage of households with at least one LLIN, average number of LLINs per household and LLIN brand. Independent variables considered for associated with LLIN use were age, gender and sub-county.

### Data collection

All survey data collection team members were trained including didactic sessions on survey objectives, methodology and questionnaires, and thereafter conducted role plays and mock interviews with a pilot field testing session which was used to provide feedback to trainees. The teams used hand-held computer tablets programmed using Surveybe software (EDI, version 5.8.24, UK).

### Data analysis

Stata version 14 (Statcorp, College Station, Texas, USA) was used for all data analysis. The distributions of study baseline characteristics (status of de-facto member, age, gender, pregnancy status, women aged 15–45, area of residence and sub-county), estimations of LLIN ownership, access and use were presented as frequencies with respective proportions and accompanying ninety-five percent confidence intervals. A chi-square test was used to estimate if the differences in proportions in LLIN ownership, access and used were statistically significant between the first survey, before mass distribution and second survey, after mass distribution.

A multivariable logistic regression model was used to assess for the factors influencing the use of LLINs in the population to derive first the crude and then the adjusted odds ratio with its respective confidence interval. In all analyses, a P-value of <0.05 was taken as statistically significant.

### Ethical considerations

The study was approved by the Makerere University School of Biomedical Sciences Higher Degrees Research and Ethics Committee (SBS-HDREC) and the Uganda National Council for Science and Technology (UNCST) ethics bodies. All participants interviewed gave their informed consent to participate in this survey and for the information derived to be published. Assent was sought for all participants aged between 15–17 years and for those that agreed, consent was obtained from their household head/guardian. The data used in this analysis was anonymous with no individual names of participants captured.

## Results section

### Baseline characteristics

There were slightly more households (173 households) enumerated in the second survey as compared to the first survey (Fig 1) with 601 households (200 in both Kapujan and Toroma and 201 in Magoro) included in the first round while 607 households (204 in both Kapujan and Magoro and 199 in Toroma) were included in the second round. However, approximately the same proportion of households were selected from both surveys; 601/8,330 (7.21%) in the first survey versus 607/8,503 (7.14%) in the second survey.

Additionally, in both surveys, there was an even distribution of the selected households across the three sub-counties as shown in maps of Fig 2.

A total of 3,045 and 2,894 de-facto household members were included in the baseline and round two survey respectively (Table 1). In both surveys, over 99% were usual residents, over 90% were from a rural setting, children under 5 and women aged 15–45 contributed approximately 20% of the population while pregnant women were slightly more in the baseline survey at 2.00% compared to the round two survey at 1.69%

**Table 1:** Baseline individual level characteristics of de-facto household members.

Characteristics	Survey timing	
	Before mass distribution N = 3,045	After mass distribution N = 2,894
	n(%)	n(%)
De-facto population		
Residents	3,040(99.84)	2,890(99.86)
Visitors	5(0.16)	4(0.14)
Age group (years)		
0–5	677(22.23)	614(21.22)
6–14	883(29.00)	854(29.51)
15–45	1,072(35.21)	1,024(35.38)
>45	413(13.56)	402(13.89)
Gender		
Male	1,476(48.47)	1,412(48.79)
Female	1,569(51.53)	1,482(51.21)
Pregnant women	61(2.00)	49(1.69)
Women aged 15–45	606(19.93)	586(20.25)
Area of residence		
Urban	38(6.87)	27(5.44)
Rural	515(93.13)	469(94.56)
Sub-county		
Kapujan	1,010(33.17)	985(34.04)
Magoro	1,064(34.94)	1,000(34.55)
Toroma	971(31.89)	909(31.41)

### Household ownership of LLINs

The percentage of households with at least one LLIN significantly rose by 8.37 percent points from baseline at 87.19% to 95.56% after the mass distribution campaign (Table 2). The percentage of households with at least one net for every two persons (households that achieved universal coverage) who stayed in the household last night was much lower at 60.57% at baseline and rose modestly by a statistically significant 9.78 percent points to 70.35% after the campaign.

**Table 2:** Household ownership of LLINs before and after mass distribution.

LLIN ownership indicators	Survey timing					
	Before mass distribution Households, N = 601		After mass distribution Households, N = 607			
	n	Percent (95%CI)	n	Percent (95% CI)	Percent difference	Chi p-value
Percentage of households with at least one LLIN	524	87.19(84.24–89.76)	580	95.56(93.59–97.04)	8.37	**0.001**
Percentage of households with at least one net for every two persons who stayed in the household last night	364	60.57(56.53–64.50)	427	70.35(66.54–73.96)	9.78	**0.001**
Average number of LLINs per household						
0 nets	83	13.81(11.15–16.82)	21	3.46(2.15–5.24)	-10.35	
1 net	157	26.12(22.65–29.83)	79	13.01(10.44–15.95)	-13.11	
2 nets	163	27.12(23.60–30.87)	163	26.85 (23.36–30.57)	0.27	
3 nets	99	16.47(13.59–19.68)	155	25.53(22.11–29.20)	9.06	
4 nets	48	7.99(5.95–10.45)	93	15.32(12.55–18.43)	7.33	
5+ nets	57	9.48(7.3–12.11)	90	14.82(12.09–17.91)	5.34	**0.001**

Similarly, the trend of the average number of LLIN per household significantly increased from the baseline with a greater percentage of households owning more than 3 LLIN nets after the campaign.

Over 85% of LLINs owned both at baseline and after the mass campaign were of the Permanent brand, however the largest source of LLINs at baseline was government health facility (39.88%) while the mass campaign was the largest source of LLINs owned during the second survey assessment (Table 3).

**Table 3:** Brand and source of LLINs.

Characteristics of LLINs	Survey timing	
	Before mass distribution N = 1,770	After mass distribution N = 1,750
**LLIN Brand**		
Permanent	1,479(87.51)*	**1,638(93.60)**
Duranet	0	8(0.46)
Olyset	0	13(0.74)
DK brand	0	86(4.91)
Other brand	211(12.49)	5(0.28)
**Source of LLINS**		
Government hospital	319(18.02)	6(0.34)^#^
Government health center	674(38.08)	78(4.46)
PNFP/NGO hospital	51(2.88)	0
PNFP/NGO health center	15(0.85)	3(0.17)
Private clinic	0	0
Private pharmacy	9(0.51)	0
Shop	64(3.62)	18(1.03)
Open market	443(25.03)	11(0.63)
Hawker	5(0.28)	0
Campaign	169(9.55)	1,241(70.91)
Church	5(0.28)	93(5.31)
Other	8(0.45)	35(20.00)
Don’t know	8(0.45)	145(8.29)

*missing 80

^#^missing 120

### Access to LLINs

**Fig 3:**
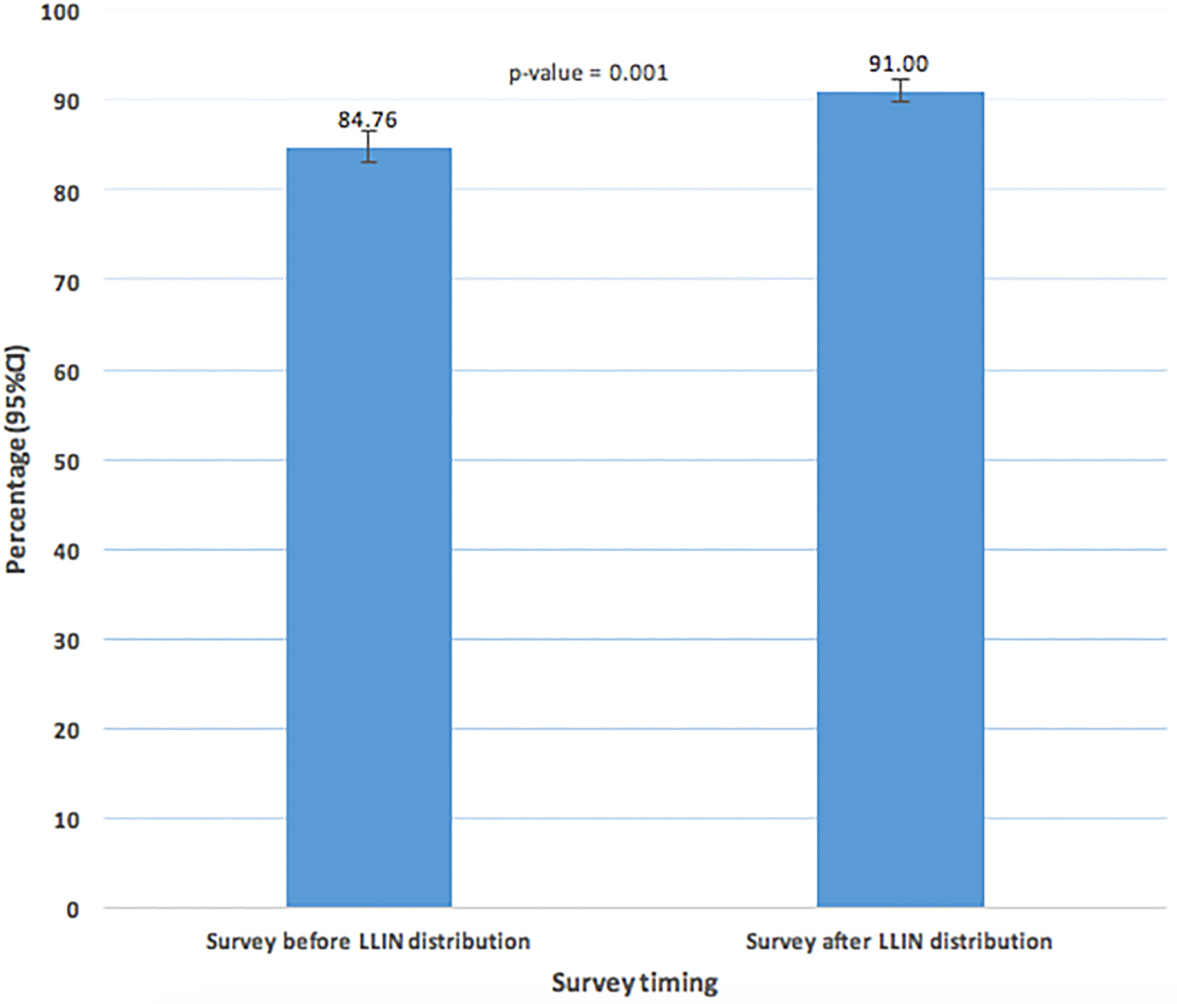
Percentage of the population with access to an LLIN with in their household.

A total of 3,045 de-facto individuals were included from the first survey households while 2,894 were from the second survey. At baseline 84.76%(82.99–86.52) of the surveyed population had access to an LLIN and this rose to 91.00% (90.33–92.81) after the mass distribution campaign, a statistically significant 6.24 percent points increase, p = 0.001 (Fig 3).

### Use of LLINs

Overall, the percentage of the de-facto household population that slept under an LLIN the last night was at 56.85% (55.06–58.62) at baseline and rose by a significant 24.87 percent points to 81.72% (80.26–83.11) after the mass campaign (Table 4). Similarly, the percentage of children under 5 years and pregnant women that slept under an LLIN the night before the survey significantly rose by 26.36 percent points (baseline = 53.77% [49.92–57.57] vs post campaign = 80.13%[76.75–83.21]) and by 19.31 percent points (baseline = 70.49% [57.43–81.48]vs post campaign = 89.80%[77.78–96.60]) respectively. The percentage of women aged 15 to 45 years that slept under an LLIN the last night also significantly rose by 17.60 percent points from the baseline estimates at 65.51%(61.58–69.30) to 83.11% (79.82–86.05) after the mass campaign. The majority of LLINs owned by the households were used the night before the survey (baseline = 99.33% vs post campaign = 92.34%).

**Table 4:** Use of LLINs the night before survey before and after mass distribution.

LLIN usage indicators	Survey timing					
	Before mass distribution		After mass distribution			
	n/N	Percent (95%CI)	n/N	Percent (95% CI)	Percent difference	Chi p-value
Percentage of LLIN used by anyone last night	1,679/1,690	99.33(98.83–99.67)	1,616/1,750	92.34(90.99–93.54)	-6.99	0.001
Percentage of household population that slept under an LLIN the last night	1,731/3,045	56.85(55.06–58.62)	2,365/2,894	81.72(80.26–83.11)	24.87	0.001
Percentage of children under 5 years that slept under an LLIN the last night	364/677	53.77(49.92–57.57)	492/614	80.13(76.75–83.21)	26,36	0.001
Percentage of pregnant women that slept under an LLIN the last night	43/61	70.49(57.43–81.48)	44/49	89.80(77.78–96.60)	19.31	0.011
Percentage of women aged 15 to 45 years that slept under an LLIN the last night	397/606	65.51(61.58–69.30)	487/586	83.11(79.82–86.05)	17.60	0.001

### Factors associated with LLIN use after mass distribution

These results presented only considered LLIN use after the mass campaign. Table 5 shows that the use of LLINs among adults older than 45 years is significantly twice as much when compared to children under 5 years. Individuals from Kapujan sub-county use LLINs more than any of the other two sub-counties, however, this difference was only significant when compared to residents in Magoro sub-county (Adjusted OR = 0.70 [0.55–0.88];p = 0.003, reference group is Kapujan). The differences in proportion observed under the gender variable were not statically significant.

**Table 5:** Individual level factors associated with LLIN use after mass distribution.

Individual level variable	LLIN use night before survey				
	Yes N = 2,368	No N = 526	Crude OR (95% CI)	Adjusted OR^#^ (95% CI)	p-value
Age group (years)					
0–5	492(80.13)	122(19.87)	1	1	
6–14	665(77.87)	189(22.13)	0.87(0.67–1.12)	0.86(0.67–1.11)	0.263
15–45	851(83.11)	173(16.89)	1.23(0.95–1.59)	1.23(0.95–1.60)	0.121
> 45	360(89.55)	42(10.45)	2.12(1.46–3.09)	2.12(1.45–3.08)	**0.001**
Gender					
Male	1,156(81.87)	256(18.13)			
Female	1,212(81.78)	270(18.22)	0.99(0.82–1.20)	0.96(0.79–1.16)	0.677
Sub-county					
Kapujan	831(84.37)	154(15.63)	1	1	
Magoro	792(79.20)	208(20.80)	0.70(0.56–0.88)	0.70(0.55–0.88)	**0.003**
Toroma	744(81.85)	165(18.15)	0.83(0.65–1.06)	0.82(0.64–1.04)	0.118

^#^Adjusted for age-group, gender and sub-county

## Discussion

Almost all the households surveyed in the study setting owned at least one LLIN with a significantly greater proportion having more than three LLINs when compared to the baseline, after the most recent mass distribution campaign. However, the percentage of households that achieved universal coverage (with at least one net for every two persons who stayed in the household last night) rose by approximately 10 percent points from the baseline percentage of sixty percent to seventy percent, an estimate still below the NMCP target of eighty-five percent [7]. Nonetheless, this is an indication of a leap towards the right direction to fulfillment of this target, especially when compared to the 2014 Malaria Indicator estimate at sixty-two percent, which was conducted one year after the 2013 mass campaign [11]. Indeed, Uganda reports a higher proportions of households achieving universal coverage after a mass distribution campaign when compared to other Sub-Saharan countries [14–16].

Similarly, the population surveyed with access to an LLIN (assuming that one LLIN covers two persons), one of the most important indicators used to measure the success of mass campaigns, significantly rose from eighty-five percent to ninety-one percent. Even though the WHO and NMCP have differing estimate considerations for a successful campaign, with the WHO target of at least eighty percent of people that have access to LLINs in the households [6] and the NMCP at eighty-five percent [7], this study has clearly shown that both these targets have been surpassed. This achievement could be interpreted in the context that the baseline percentage was already at or above the set target before the mass campaign, however, it is more likely that the baseline estimates were of the older worn out and less effective LLINs, while the most recent campaign provided new and more effective LLINs as recommended by the WHO [5]. This is important especially when the mode of action of LLINs for malaria prevention is taken into consideration. LLINs prevent malaria transmission by serving as not only physical barriers between mosquito vectors and individual users but also through the pyrethroid insecticide that is repellent and toxic to mosquitoes whose efficacy reduces with time and therefore LLINs that are 3 years or more are not as effective in prevention of malaria [5,17–19].

Of greater importance to a successful mass campaign is that the household population uses the LLINs to prevent malaria especially among children under five years and pregnant, who are the most at risk of acquiring malaria [4,7]. Over eighty percent of all de-facto household members surveyed slept under an LLIN the night before, a similar proportion was reported for children under five years and an even greater proportion (eighty-nine percent) among pregnant women, all above the eighty percent WHO set target for success [6]. Nonetheless, this achievement was also considered against the NMCP target at eighty-five percent of the population using an LLIN [7] and in this context, the percentage of pregnant women who slept under an LLIN the night before was the only category to surpass this target.

Although, the mass campaign exercise was to a great extent a successful one, based on the WHO indicators, it is important to note that there was a higher percentage of the population that could access an LLIN (ninety-one percent) as compared to the lesser proportion that used one (eighty-two percent), a finding that has been previously reported in other studies [20–23]. This is an indication that there still exists behavioral change gaps and challenges affecting LLIN usage in this setting. Therefore the NMCP and stakeholders should not only aim at achieving universal coverage by routinely distributing more LLINs, but equally focus on behavioral change communication to encourage bed net usage as the ultimate goal for malaria prevention [5,7]. Unfortunately, this study was not designed to robustly answer questions on factors associated with LLIN use, however one was noted. Residents in Kapujan sub-country were significantly more likely to use an LLIN as compared to those in Magoro. To give this scenario context, the primary study from which this project is part, has Kapujan residents assigned to MDA and IRS for malaria prevention unlike residents in Magoro, which is the control sub-county. The noticed difference could be due to the sensitization campaigns in Kapujan on LLIN use as part of the broader message during the study interventions implementation. This indirectly points to the importance of continuous mass sensitization and behavioral change communication in prevention of malaria including the use of LLINs [24–26].

These findings are relevant to guiding future LLIN campaigns even though the study covered a small region of the country and therefore smaller sample size when compared to the large Malaria Indicator Surveys. This is one of the first robust before and after studies exploring the effectiveness of mass campaign approach to achieve universal coverage in our setting. The study strength lies in the identical design and implementation of activities with the Uganda MIS surveys [11–12], including the random selection of households to minimize selection bias, and therefore the same methodology was used to derive these estimates and can be comparable by setting.

### Conclusion

The LLIN mass campaign was successful in ensuring that at least eighty-five prevent of the population had access to an LLIN and over eighty percent of these slept under an LLIN the night before. However, there are areas for improvement including increasing the percentage of households that achieve universal coverage and also the wide gap between LLIN access and use points to a behavioral change communication gap. The consideration of these recommendations during the planning phases of the future LLIN mass campaigns will be very useful in tailoring efforts towards the current deficient areas instead of merely repeating the routine activities of mass campaigns.

## Supporting information

S1 File 2014 Uganda MIS_Household questionnaire. (PDF)

S2 File 2014 Uganda MIS_Women’s questionnaire. (PDF)

S3 File LLIN data excel file. (XLSX)
