# Supplementary material for: Long lasting insecticidal bed nets ownership, access and use in a high malaria transmission setting before and after a mass distribution campaign in Uganda
Source: PLoS One. 2018 Jan 18;13(1):e0191191. doi: 10.1371/journal.pone.0191191 (PMC5773208; doi:10.1371/journal.pone.0191191)
Supplement: S1 File — (PDF) [file pone.0191191.s001.pdf]

2014 UGANDA MALARIA INDICATOR SURVEY
HOUSEHOLD QUESTIONNAIRE

UGANDA
IDRC/MOH/UBOS

FORMATTING DATE: 20 November 2014
LANGUAGE DATE: 19 November 2014

IDENTIFICATION (1)

REGION ____________________

DISTRICT ____________________

COUNTY ____________________

SUBCOUNTY/TOWN ____________________

PARISH/LC2 NAME ____________________

EA NAME ____________________

UMIS NUMBER . . . .

URBAN=1, PERI URBAN=2, RURAL=3 . . . .

NAME OF HEAD OF HOUSEHOLD ____________________

HOUSEHOLD NUMBER . . . .

HOUSEHOLD SAMPLE NUMBER . . . .

INTERVIEWER VISITS

	1	2	3	FINAL VISIT
DATE	________	________	________	DAY<br>MONTH<br>YEAR
INTERVIEWER'S NAME	________	________	________	INT. NUMBER
RESULT*	________	________	________	RESULT
NEXT VISIT: DATE	________	________		TOTAL NUMBER OF VISITS
TIME	________	________		

*RESULT CODES:
1 COMPLETED
2 NO HOUSEHOLD MEMBER AT HOME OR NO COMPETENT RESPONDENT AT HOME AT TIME OF VISIT
3 ENTIRE HOUSEHOLD ABSENT FOR EXTENDED PERIOD OF TIME
4 POSTPONED
5 REFUSED
6 DWELLING VACANT OR ADDRESS NOT A DWELLING
7 DWELLING DESTROYED
8 DWELLING NOT FOUND
9 OTHER ____________________
(SPECIFY)

TOTAL PERSONS IN HOUSEHOLD

TOTAL ELIGIBLE WOMEN

TOTAL ELIGIBLE CHILDREN

LINE NO. OF RESPONDENT TO HOUSEHOLD QUESTIONNAIRE

LANGUAGE OF THE QUESTIONNAIRE 7

LANGUAGE USED IN THE INTERVIEW . . . .

NATIVE LANGUAGE OF RESPONDENT . . . .

TRANSLATOR USED (NOT AT ALL=1; SOMETIMES=2; ALL THE TIME=3) . . . . . .

LANGUAGE OF THE QUESTIONNAIRE **English**

LANGUAGE USED:

1 ATESO-KARAMOJONG
2 LUGANDA
3 LUGBARA
4 LUO
5 RUNYANKORE-RUKIGA
6 RUNYORO-RUTORO
7 ENGLISH
8 OTHER ____________
(SPECIFY)

SUPERVISOR NAME ____________

OFFICE EDITOR

KEYED BY

THIS PAGE IS INTENTIONALLY BLANK

INFORMED CONSENT

Hello. My name is _______________________________________. I am working with the Ministry of Health. We are conducting a survey about malaria all over Uganda. The information we collect will help the government to plan health services. Your household was selected for the survey. I would like to ask you some questions about your household. The questions usually take about 15 to 20 minutes. All of the answers you give will be confidential and will not be shared with anyone other than members of our survey team. You don't have to be in the survey, but we hope you will agree to answer the questions since your views are important. If I ask you any question you don't want to answer, just let me know and I will go on to the next question or you can stop the interview at any time.
In case you need more information about the survey, you may contact the person listed on this card.

GIVE CARD WITH CONTACT INFORMATION

Do you have any questions?
May I begin the interview now?

SIGNATURE OF INTERVIEWER: ____________________ DATE: ____________________

RESPONDENT AGREES TO BE INTERVIEWED . . . 1 ↓ RESPONDENT DOES NOT AGREE TO BE INTERVIEWED . . . 2 → END

START TIME:

HOURS . . . . [ ][ ]

MINUTES . . . . [ ][ ]

HOUSEHOLD SCHEDULE

LINE NO.	USUAL RESIDENTS AND VISITORS	RELATIONSHIP TO HEAD OF HOUSEHOLD	SEX	RESIDENCE		AGE	WOMEN AGE 15-49	CHILDREN AGE 0-5
1	2	3	4	5	6	7	8	9
	Please give me the names of the persons who usually live in your household and guests of the household who stayed here last night, starting with the head of the household.<br><br>AFTER LISTING THE NAMES AND RECORDING THE RELATIONSHIP AND SEX FOR EACH PERSON, ASK QUESTIONS 2A-2C TO BE SURE THAT THE LISTING IS COMPLETE.<br><br>THEN ASK APPROPRIATE QUESTIONS IN COLUMNS 5-9 FOR EACH PERSON.	What is the relationship of (NAME) to the head of the household?<br><br>SEE CODES BELOW.	Is (NAME) male or female?	Does (NAME) usually live here?	Did (NAME) stay here last night?	How old is (NAME)?<br><br>IF 95 OR MORE, RECORD '95'.	CIRCLE LINE NUMBER OF ALL WOMEN AGE 15-49	CIRCLE LINE NUMBER OF ALL CHILDREN AGE 0-5
			M F	Y N	Y N	IN YEARS		
01			1 2	1 2	1 2		01	01
02			1 2	1 2	1 2		02	02
03			1 2	1 2	1 2		03	03
04			1 2	1 2	1 2		04	04
05			1 2	1 2	1 2		05	05
06			1 2	1 2	1 2		06	06
07			1 2	1 2	1 2		07	07
08			1 2	1 2	1 2		08	08
09			1 2	1 2	1 2		09	09
10			1 2	1 2	1 2		10	10

CODES FOR Q. 3: RELATIONSHIP TO HEAD OF HOUSEHOLD

01 = HEAD
02 = WIFE OR HUSBAND
03 = SON OR DAUGHTER
04 = SON-IN-LAW OR DAUGHTER-IN-LAW
05 = GRANDCHILD
06 = PARENT
07 = PARENT-IN-LAW
08 = BROTHER OR SISTER
09 = OTHER RELATIVE
10 = ADOPTED/FOSTER/ STEPCHILD
11 = NOT RELATED
98 = DON'T KNOW

LINE NO.	USUAL RESIDENTS AND VISITORS	RELATIONSHIP TO HEAD OF HOUSEHOLD	SEX	RESIDENCE		AGE	WOMEN AGE 15-49	CHILDREN AGE 0-5
1	2	3	4	5	6	7	8	9
	Please give me the names of the persons who usually live in your household and guests of the household who stayed here last night, starting with the head of the household.<br><br>AFTER LISTING THE NAMES AND RECORDING THE RELATIONSHIP AND SEX FOR EACH PERSON, ASK QUESTIONS 2A-2C TO BE SURE THAT THE LISTING IS COMPLETE.<br><br>THEN ASK APPROPRIATE QUESTIONS IN COLUMNS 5-9 FOR EACH PERSON.	What is the relationship of (NAME) to the head of the household?<br><br>SEE CODES BELOW.	Is (NAME) male or female?	Does (NAME) usually live here?	Did (NAME) stay here last night?	How old is (NAME)?<br><br>IF 95 OR MORE, RECORD '95'.	CIRCLE LINE NUMBER OF ALL WOMEN AGE 15-49	CIRCLE LINE NUMBER OF ALL CHILDREN AGE 0-5
			M F	Y N	Y N	IN YEARS		
11		☐☐	1 2	1 2	1 2	☐☐	11	11
12		☐☐	1 2	1 2	1 2	☐☐	12	12
13		☐☐	1 2	1 2	1 2	☐☐	13	13
14		☐☐	1 2	1 2	1 2	☐☐	14	14
15		☐☐	1 2	1 2	1 2	☐☐	15	15
16		☐☐	1 2	1 2	1 2	☐☐	16	16
17		☐☐	1 2	1 2	1 2	☐☐	17	17
18		☐☐	1 2	1 2	1 2	☐☐	18	18
19		☐☐	1 2	1 2	1 2	☐☐	19	19
20		☐☐	1 2	1 2	1 2	☐☐	20	20

TICK HERE IF CONTINUATION SHEET USED ☐

2A) Just to make sure that I have a complete listing: are there any other persons such as small children or infants that we have not listed? YES ☐ → ADD TO TABLE NO ☐

2B) Are there any other people who may not be members of your family, such as domestic servants, lodgers, or friends who usually live here? YES ☐ → ADD TO TABLE NO ☐

2C) Are there any guests or temporary visitors staying here, or anyone else who stayed here last night, who have not been listed? YES ☐ → ADD TO TABLE NO ☐

CODES FOR Q.3: RELATIONSHIP TO HEAD OF HOUSEHOLD

01 = HEAD
02 = WIFE OR HUSBAND
03 = SON OR DAUGHTER
04 = SON-IN-LAW OR DAUGHTER-IN-LAW
05 = GRANDCHILD
06 = PARENT
07 = PARENT-IN-LAW
08 = BROTHER/SISTER
09 = OTHER RELATIVE
10 = ADOPTED/FOSTER/STEPCHILD
11 = NOT RELATED
98 = DON'T KNOW

HOUSEHOLD CHARACTERISTICS

NO.	QUESTIONS AND FILTERS	CODING CATEGORIES	SKIP
101	What is the main source of drinking water for members of your household?	PIPED WATER<br>PIPED INTO DWELLING . . . . 11<br>PIPED TO YARD/PLOT . . . . 12<br>PUBLIC TAP/STANDPIPE . . . . 13<br>BOREHOLE IN YARD/PLOT . . . . 21<br>PUBLIC BOREHOLE . . . . 22<br>DUG WELL<br>PROTECTED WELL . . . . 31<br>UNPROTECTED WELL . . . . 32<br>WATER FROM SPRING<br>PROTECTED SPRING . . . . 41<br>UNPROTECTED SPRING . . . . 42<br>GRAVITY FLOW SCHEME . . . . 43<br>RAINWATER . . . . 51<br>TANKER TRUCK . . . . 61<br>VENDOR . . . . 62<br>CART WITH SMALL TANK . . . . 71<br>SURFACE WATER (RIVER/DAM/<br>LAKE/POND/STREAM/CANAL/<br>IRRIGATION CHANNEL) . . . . 81<br>BOTTLED WATER . . . . 91<br><br>OTHER ______________________ 96<br>(SPECIFY)	11, 12 → 104<br>21 → 104<br>51 → 104
102	Where is that water source located?	IN OWN DWELLING . . . . 1<br>IN OWN YARD/PLOT . . . . 2<br>ELSEWHERE 3	1, 2 → 104
103	How long does it take to go there, get water, and come back?	MINUTES . . . . [ ][ ][ ]<br><br>DON'T KNOW 998	
104	What kind of toilet facility do members of your household usually use?	FLUSH OR POUR FLUSH TOILET<br>FLUSH TO PIPED SEWER<br>SYSTEM . . . . 11<br>FLUSH TO SEPTIC TANK . . . . 12<br>FLUSH TO PIT LATRINE . . . . 13<br>FLUSH TO SOMEWHERE ELSE . . . . 14<br>FLUSH, DON'T KNOW WHERE . . . . 15<br>PIT LATRINE<br>VENTILATED IMPROVED<br>PIT LATRINE . . . . 21<br>COVERED PIT LATRINE WITH SLAB . . . 22<br>COVERED PIT LATRINE WITHOUT SLAB/<br>OPEN PIT . . . . 23<br>UNCOVERED PIT LATRINE WITH SLAB . . 24<br>UNCOVERED PIT LATRINE<br>WITHOUT SLAB . . . . 25<br>COMPOSTING TOILET (ECOSAN) . . . . 31<br>BUCKET TOILET . . . . 41<br>HANGING TOILET/HANGING<br>LATRINE . . . . 51<br>NO FACILITY/BUSH/FIELD/BAGS/BUCKET . . 61<br><br>OTHER ______________________ 96<br>(SPECIFY)	61 → 107
105	Do you share this toilet facility with other households?	YES . . . . 1<br>NO 2	2 → 107
106	How many households use this toilet facility?	NO. OF HOUSEHOLDS<br>IF LESS THAN 10 . . . . [0][ ]<br><br>10 OR MORE HOUSEHOLDS . . . . 95<br>DON'T KNOW 98	

NO.	QUESTIONS AND FILTERS	CODING CATEGORIES			SKIP
107	Does your household have:		YES	NO	
	a) Electricity?	a) ELECTRICITY	1	2	
	b) A radio?	b) RADIO	1	2	
	c) A cassette player?	c) CASSETTE PLAYER	1	2	
	d) A television?	d) TELEVISION	1	2	
	e) A mobile telephone?	e) MOBILE TELEPHONE	1	2	
	f) A non-mobile telephone?	f) NON-MOBILE TELEPHONE	1	2	
	g) A refrigerator?	g) REFRIGERATOR	1	2	
	h) A table?	h) TABLE	1	2	
	i) A chair?	i) CHAIR	1	2	
	j) A sofa set?	j) SOFA SET	1	2	
	k) A bed?	k) BED	1	2	
	l) A cupboard?	l) CUPBOARD	1	2	
	m) A clock?	m) CLOCK	1	2	
108	What type of fuel does your household mainly use for cooking?	ELECTRICITY . . . . 01<br>LPG . . . . 02<br>NATURAL GAS . . . . 03<br>BIOGAS . . . . 04<br>KEROSENE/PARAFFIN . . . . 05<br>COAL, LIGNITE . . . . 06<br>CHARCOAL . . . . 07<br>WOOD . . . . 08<br>STRAW/SHRUBS/GRASS . . . . 09<br>AGRICULTURAL CROP . . . . 10<br>ANIMAL DUNG . . . . 11<br>NO FOOD COOKED<br>IN HOUSEHOLD . . . . 95<br>OTHER ______________________ 96<br>(SPECIFY)			
109	MAIN MATERIAL OF THE FLOOR.<br><br>RECORD OBSERVATION.	NATURAL FLOOR<br>EARTH/SAND . . . . 11<br>SAND AND DUNG . . . . 12<br>RUDIMENTARY FLOOR<br>WOOD PLANKS . . . . 21<br>PALM . . . . 22<br>FINISHED FLOOR<br>PARQUET/POLISHED WOOD . . . . 31<br>MOSAIC OR TILE . . . . 33<br>CEMENT . . . . 34<br>STONES . . . . 36<br>BRICKS . . . . 37<br>OTHER ______________________ 96<br>(SPECIFY)			
110	MAIN MATERIAL OF THE ROOF.<br><br>RECORD OBSERVATION.	NATURAL ROOFING<br>NO ROOF . . . . 11<br>THATCHED . . . . 12<br>MUD . . . . 13<br>RUDIMENTARY ROOFING<br>TIN . . . . 21<br>PALM . . . . 22<br>WOOD PLANKS . . . . 23<br>FINISHED ROOFING<br>IRON SHEETS . . . . 31<br>WOOD . . . . 32<br>CEMENT FIBER . . . . 33<br>TILES . . . . 34<br>CEMENT . . . . 35<br>ROOFING SHINGLES . . . . 36<br>ASBESTOS . . . . 37<br>OTHER ______________________ 96<br>(SPECIFY)			

NO.	QUESTIONS AND FILTERS	CODING CATEGORIES	SKIP
111	MAIN MATERIAL OF THE EXTERIOR WALLS.<br><br>RECORD OBSERVATION.	NATURAL WALLS<br>NO WALLS . . . 11<br>THATCHED/STRAW . . . 12<br>DIRT . . . 13<br>RUDIMENTARY WALLS<br>MUD AND POLES . . . 21<br>STONE WITH MUD . . . 22<br>REUSED WOOD . . . 26<br>UNBURNT BRICKS . . . 27<br>UNBURNT BRICKS WITH PLASTER . . . 28<br>UNBURNT BRICKS WITH MUD . . . 29<br>FINISHED WALLS<br>CEMENT . . . 31<br>STONE WITH LIME/CEMENT . . . 32<br>BURNT BRICKS WITH CEMENT . . . 33<br>CEMENT BLOCKS . . . 34<br>WOOD PLANKS/SHINGLES . . . 36<br><br>OTHER ______ 96<br>(SPECIFY)	
112	How many rooms in this household are used for sleeping?	ROOMS . . . [ ][ ]	
113	Does any member of this household own:<br>a) A watch?<br>b) A bicycle?<br>c) A motorcycle or motor scooter?<br>d) An animal-drawn cart?<br>e) A car or truck?<br>f) A boat with a motor?<br>g) A boat without a motor?	YES NO<br>a) WATCH . . . 1 2<br>b) BICYCLE . . . 1 2<br>c) MOTORCYCLE/SCOOTER . . . 1 2<br>d) ANIMAL-DRAWN CART . . . 1 2<br>e) CAR/TRUCK . . . 1 2<br>f) BOAT WITH MOTOR . . . 1 2<br>g) BOAT WITH NO MOTOR . . . 1 2	
114	Does any member of this household own any agricultural land?	YES . . . 1<br>NO . . . 2	 → 116
115	How many acres of agricultural land do members of this household own?<br><br>IF 95 OR MORE, CIRCLE '950'.<br>(1 DECIMAL = 00.1 ACRES)	ACRES . . . [ ][ ].[ ]<br><br>95 OR MORE ACRES . . . 950<br>DON'T KNOW . . . 998	
116	Does this household own any livestock, herds, other farm animals, or poultry?	YES . . . 1<br>NO . . . 2	 → 118
117	How many of the following animals does this household own?<br><br>IF NONE, ENTER '00'.<br>IF 95 OR MORE, ENTER '95'.<br>IF UNKNOWN, ENTER '98'.<br><br>a) Cattle?<br>b) Milk cows or bulls?<br>c) Horses, donkeys, or mules?<br>d) Goats?<br>e) Sheep?<br>f) Chickens?<br>g) Pigs?	 <br><br><br><br><br>a) CATTLE . . . [ ][ ]<br>b) COWS/BULLS . . . [ ][ ]<br>c) HORSES/DONKEYS/MULES . . . [ ][ ]<br>d) GOATS . . . [ ][ ]<br>e) SHEEP . . . [ ][ ]<br>f) CHICKENS . . . [ ][ ]<br>g) PIGS . . . [ ][ ]	

NO.	QUESTIONS AND FILTERS	CODING CATEGORIES	SKIP
118	Does any member of this household have a bank account?	YES . . . . 1<br>NO 2	
119	At any time in the past 6 months, has anyone come into your dwelling to spray the interior walls against mosquitoes?	YES . . . . 1<br>NO . . . . 2<br>DON'T KNOW 8	2, 8 → 120B
119A	How many months ago was the dwelling last sprayed?<br><br>IF LESS THAN ONE MONTH, RECORD '0' MONTHS AGO.	MONTHS AGO . . . . ☐	
120	Who sprayed the dwelling?	GOVERNMENT WORKER/PROGRAM . . . . . A<br>PRIVATE COMPANY . . . . B<br>NONGOVERNMENTAL<br>ORGANIZATION (NGO) . . . . C<br><br>OTHER ______________ X<br>(SPECIFY)<br>DON'T KNOW . . . . Z	
120A	Did you pay for your dwelling to be sprayed?	YES . . . . 1<br>NO . . . . 2<br>DON'T KNOW 8	
120B	Is there a community worker or community medicine distributor (CMD) who distributes malaria medicines in your village or community?	YES . . . . 1<br>NO . . . . 2<br>DON'T KNOW 8	2, 8 → 121
120C	Does the community health worker currently have malaria medicines available?	YES . . . . 1<br>NO . . . . 2<br>DON'T KNOW 8	
121	Does your household have any mosquito nets that can be used while sleeping?	YES . . . . 1<br>NO 2	2 → 201
122	How many mosquito nets does your household have?<br><br>IF 7 OR MORE NETS, RECORD '7'.	NUMBER OF NETS . . . . ☐	

		NET #1	NET #2	NET #3
123	ASK THE RESPONDENT TO SHOW YOU ALL THE NETS IN THE HOUSEHOLD<br><br>IF MORE THAN 3 NETS, USE ADDITIONAL QUESTIONNAIRE(S).	OBSERVED . . . . . 1<br>NOT OBSERVED . . . 2	OBSERVED . . . . . 1<br>NOT OBSERVED . . . 2	OBSERVED . . . . . 1<br>NOT OBSERVED . . . 2
124	How many months ago did your household get the mosquito net?<br><br>IF LESS THAN ONE MONTH AGO, RECORD '00'.	MONTHS AGO . . . [ ][ ]<br><br>MORE THAN 36 MONTHS AGO . . . 95<br><br>NOT SURE 98	MONTHS AGO . . . [ ][ ]<br><br>MORE THAN 36 MONTHS AGO . . . 95<br><br>NOT SURE 98	MONTHS AGO . . . [ ][ ]<br><br>MORE THAN 36 MONTHS AGO . . . 95<br><br>NOT SURE 98
124A	Where did you get the mosquito net from?	PUBLIC SECTOR (GOV'T)<br>GOV'T HOSPITAL . . 01<br>GOV'T HEALTH CENTER . . . . 02<br>PUBLIC SECTOR(PNFP/NGO)<br>HOSPITAL . . . . . 03<br>HEALTH CENTER . 04<br>PRIVATE MEDICAL SECTOR<br>PRIVATE HOSPITAL/ CLINIC . . . . 05<br>PHARMACY . . . . . 06<br>OTHER SOURCE<br>SHOP . . . . 07<br>OPEN MARKET . . . 08<br>HAWKER . . . . 09<br>CAMPAIGN . . . . . 10<br>CHURCH . . . . 11<br>OTHER . . . . 96<br>DOES NOT KNOW . . . 98	PUBLIC SECTOR (GOV'T)<br>GOV'T HOSPITAL . . 01<br>GOV'T HEALTH CENTER . . . . 02<br>PUBLIC SECTOR(PNFP/NGO)<br>HOSPITAL . . . . . 03<br>HEALTH CENTER . 04<br>PRIVATE MEDICAL SECTOR<br>PRIVATE HOSPITAL/ CLINIC . . . . 05<br>PHARMACY . . . . . 06<br>OTHER SOURCE<br>SHOP . . . . 07<br>OPEN MARKET . . . 08<br>HAWKER . . . . 09<br>CAMPAIGN . . . . . 10<br>CHURCH . . . . 11<br>OTHER . . . . 96<br>DOES NOT KNOW . . . 98	PUBLIC SECTOR (GOV'T)<br>GOV'T HOSPITAL . . 01<br>GOV'T HEALTH CENTER . . . . 02<br>PUBLIC SECTOR(PNFP/NGO)<br>HOSPITAL . . . . . 03<br>HEALTH CENTER . 04<br>PRIVATE MEDICAL SECTOR<br>PRIVATE HOSPITAL/ CLINIC . . . . 05<br>PHARMACY . . . . . 06<br>OTHER SOURCE<br>SHOP . . . . 07<br>OPEN MARKET . . . 08<br>HAWKER . . . . 09<br>CAMPAIGN . . . . . 10<br>CHURCH . . . . 11<br>OTHER . . . . 96<br>DOES NOT KNOW . . . 98
124B	CHECK 124A. THE NET WAS OBTAINED THROUGH THE CAMPAIGN?	CODE '10' CIRCLED [ ]<br>CODE '10' NOT CIRCLED [ ] (SKIP TO 125)	CODE '10' CIRCLED [ ]<br>CODE '10' NOT CIRCLED [ ] (SKIP TO 125)	CODE '10' CIRCLED [ ]<br>CODE '10' NOT CIRCLED [ ] (SKIP TO 125)
124C	What is the campaign's date?	DAY . . . . [ ][ ]<br>MONTH . . . . . [ ][ ]<br>YEAR [ ][ ][ ][ ]<br>DON'T KNOW 98	DAY . . . . [ ][ ]<br>MONTH . . . . . [ ][ ]<br>YEAR [ ][ ][ ][ ]<br>DON'T KNOW 98	DAY . . . . [ ][ ]<br>MONTH . . . . . [ ][ ]<br>YEAR [ ][ ][ ][ ]<br>DON'T KNOW 98

		NET #1	NET #2	NET #3
125	OBSERVE OR ASK THE BRAND/ TYPE OF MOSQUITO NET.<br><br>IF BRAND IS UNKNOWN AND YOU CANNOT OBSERVE THE NET, SHOW PICTURES OF TYPICAL NET TYPES/BRANDS	LONG-LASTING INSECTICIDE-TREATED NET (LLIN)<br>PERMANET . . . . . 11<br>DURANET . . . . . 12<br>INTERCEPTOR . . 13<br>NETPROTECT . . 14<br>OLYSET . . . . 15<br>DAWNET . . . . 16<br>ICONLIFE . . . . . 17<br>YORKOOL . . . . . 18<br>DK BRAND . . . . . 19<br>OTHER ________ 20<br>(SPECIFY)<br>(SKIP TO 128)<br><br>OTHER BRAND . . . 96<br>DK BRAND 98	LONG-LASTING INSECTICIDE-TREATED NET (LLIN)<br>PERMANET . . . . . 11<br>DURANET . . . . . 12<br>INTERCEPTOR . . 13<br>NETPROTECT . . 14<br>OLYSET . . . . 15<br>DAWNET . . . . 16<br>ICONLIFE . . . . . 17<br>YORKOOL . . . . . 18<br>DK BRAND . . . . . 19<br>OTHER ________ 20<br>(SPECIFY)<br>(SKIP TO 128)<br><br>OTHER BRAND . . . 96<br>DK BRAND 98	LONG-LASTING INSECTICIDE-TREATED NET (LLIN)<br>PERMANET . . . . . 11<br>DURANET . . . . . 12<br>INTERCEPTOR . . 13<br>NETPROTECT . . 14<br>OLYSET . . . . 15<br>DAWNET . . . . 16<br>ICONLIFE . . . . . 17<br>YORKOOL . . . . . 18<br>DK BRAND . . . . . 19<br>OTHER ________ 20<br>(SPECIFY)<br>(SKIP TO 128)<br><br>OTHER BRAND . . . 96<br>DK BRAND 98
126	Since you got the net, was it ever soaked or dipped in a liquid to kill or repel mosquitoes?	YES . . . . 1<br>NO . . . . 2<br>(SKIP TO 128)<br>NOT SURE 8	YES . . . . 1<br>NO . . . . 2<br>(SKIP TO 128)<br>NOT SURE 8	YES . . . . 1<br>NO . . . . 2<br>(SKIP TO 128)<br>NOT SURE 8
127	How many months ago was the net last soaked or dipped?<br><br>IF LESS THAN ONE MONTH AGO, RECORD '00'.	MONTHS AGO . . . [ ][ ]<br><br>MORE THAN 24 MONTHS AGO . . . 95<br><br>NOT SURE 98	MONTHS AGO . . . [ ][ ]<br><br>MORE THAN 24 MONTHS AGO . . . 95<br><br>NOT SURE 98	MONTHS AGO . . . [ ][ ]<br><br>MORE THAN 24 MONTHS AGO . . . 95<br><br>NOT SURE 98
128	Did anyone sleep under this mosquito net last night?	YES . . . . 1<br>(SKIP TO 129)<br>NO . . . . 2<br>NOT SURE . . . . . 8<br>(SKIP TO 130)	YES . . . . 1<br>(SKIP TO 129)<br>NO . . . . 2<br>NOT SURE . . . . . 8<br>(SKIP TO 130)	YES . . . . 1<br>(SKIP TO 129)<br>NO . . . . 2<br>NOT SURE . . . . . 8<br>(SKIP TO 130)
128A	What are some of the reasons why this net was not used?	TOO HOT . . . . A<br>DON'T LIKE SMELL . . B<br>NO MOSQUITOES . . C<br>NET TOO OLD/TOO MANY HOLES . . D<br>NET NOT HUNG . . E<br>OTHER__________ X<br>(SPECIFY)<br>DON'T KNOW . . . . . Z<br>(ALL SKIP TO 130)	TOO HOT . . . . A<br>DON'T LIKE SMELL . . B<br>NO MOSQUITOES . . C<br>NET TOO OLD/TOO MANY HOLES . . D<br>NET NOT HUNG . . E<br>OTHER__________ X<br>(SPECIFY)<br>DON'T KNOW . . . . . Z<br>(ALL SKIP TO 130)	TOO HOT . . . . A<br>DON'T LIKE SMELL . . B<br>NO MOSQUITOES . . C<br>NET TOO OLD/TOO MANY HOLES . . D<br>NET NOT HUNG . . E<br>OTHER__________ X<br>(SPECIFY)<br>DON'T KNOW . . . . . Z<br>(ALL SKIP TO 130)

		NET #1	NET #2	NET #3
129	Who slept under this mosquito net last night?<br><br>RECORD THE PERSON'S NAME AND LINE NUMBER FROM THE HOUSEHOLD SCHEDULE.	NAME_____________<br>LINE NO.	NAME_____________<br>LINE NO.	NAME_____________<br>LINE NO. . . . . .
		NAME_____________<br>LINE NO.	NAME_____________<br>LINE NO.	NAME_____________<br>LINE NO. . . . . .
		NAME_____________<br>LINE NO.	NAME_____________<br>LINE NO.	NAME_____________<br>LINE NO. . . . . .
		NAME_____________<br>LINE NO.	NAME_____________<br>LINE NO.	NAME_____________<br>LINE NO. . . . . .
130		GO BACK TO 123 FOR NEXT NET; OR, IF NO MORE NETS, GO TO 131.	GO BACK TO 123 FOR NEXT NET; OR, IF NO MORE NETS, GO TO 131.	GO TO 123 IN FIRST COLUMN OF A NEW QUESTIONNAIRE; OR, IF NO MORE NETS, GO TO 131.
131	RECORD THE TIME	HOURS . . . .<br>MINUTES		

HEMOGLOBIN MEASUREMENT AND MALARIA TESTING FOR CHILDREN AGE 0-5				
201	CHECK COLUMN 9 IN HOUSEHOLD SCHEDULE. RECORD THE LINE NUMBER AND NAME FOR ALL ELIGIBLE CHILDREN 0-5 YEARS IN QUESTION 202. IF MORE THAN SIX CHILDREN, USE ADDITIONAL QUESTIONNAIRE(S).			
		CHILD 1	CHILD 2	CHILD 3
202	LINE NUMBER FROM COLUMN 9<br><br>NAME FROM COLUMN 2	LINE NUMBER . . . . . [ ][ ]<br><br>NAME ________	LINE NUMBER . . . . . [ ][ ]<br><br>NAME ________	LINE NUMBER . . . . . [ ][ ]<br><br>NAME ________
203	IF MOTHER INTERVIEWED, COPY MONTH AND YEAR OF BIRTH FROM BIRTH HISTORY AND ASK DAY; IF MOTHER NOT INTERVIEWED, ASK:<br>What is (NAME'S) birth date?	DAY . . . . [ ][ ]<br>MONTH . . . . . [ ][ ]<br>YEAR [ ][ ][ ][ ]	DAY . . . . [ ][ ]<br>MONTH . . . . . [ ][ ]<br>YEAR [ ][ ][ ][ ]	DAY . . . . [ ][ ]<br>MONTH . . . . . [ ][ ]<br>YEAR [ ][ ][ ][ ]
204	CHECK 203:<br>CHILD BORN IN JANUARY 2009 OR LATER?	YES . . . . 1<br>NO . . . . 2<br>(GO TO 203 FOR NEXT CHILD OR, IF NO MORE CHILDREN, END INTERVIEW)	YES . . . . 1<br>NO . . . . 2<br>(GO TO 203 FOR NEXT CHILD OR, IF NO MORE CHILDREN, END INTERVIEW)	YES . . . . 1<br>NO . . . . 2<br>(GO TO 203 FOR NEXT CHILD OR, IF NO MORE CHILDREN, END INTERVIEW)
206	LINE NUMBER OF PARENT/ OTHER ADULT RESPONSIBLE FOR THE CHILD (FROM COLUMN 1 OF HOUSEHOLD SCHEDULE). RECORD '00' IF NOT LISTED.	LINE NUMBER . . . . . [ ][ ]	LINE NUMBER . . . . . [ ][ ]	LINE NUMBER . . . . . [ ][ ]
207	ASK CONSENT FOR ANEMIA TEST FROM PARENT/OTHER ADULT IDENTIFIED IN 206 AS RESPONSIBLE FOR CHILD.	As part of this survey, we are asking children all over the country to take an anemia test. Anemia is a serious health problem that usually results from poor nutrition, infection, or chronic disease. This survey will assist the government to develop programs to prevent and treat anemia.<br>We ask that all children born in 2009 or later take part in anemia testing in this survey and give a few drops of blood from a finger or heel. The equipment used to take the blood is clean and completely safe. It has never been used before and will be thrown away after each test.<br>The blood will be tested for anemia immediately, and the result will be told to you right away. The result will be kept strictly confidential and will not be shared with anyone other than members of our survey team.<br><br>Do you have any questions?<br>You can say yes to the test, or you can say no. It is up to you to decide.<br>Will you allow (NAME OF CHILD) to participate in the anemia test?		
208	CIRCLE THE APPROPRIATE CODE AND SIGN YOUR NAME.	GRANTED . . . . 1<br>________<br>(SIGN)<br>REFUSED . . . . 2<br>NOT PRESENT . . . . . 5<br>OTHER 6	GRANTED . . . . 1<br>________<br>(SIGN)<br>REFUSED . . . . 2<br>NOT PRESENT . . . . . 5<br>OTHER 6	GRANTED . . . . 1<br>________<br>(SIGN)<br>REFUSED . . . . 2<br>NOT PRESENT . . . . . 5<br>OTHER 6

		CHILD 1	CHILD 2	CHILD 3
	NAME FROM COLUMN 2	NAME ______________	NAME ______________	NAME ______________
209	ASK CONSENT FOR MALARIA TEST FROM PARENT/OTHER ADULT IDENTIFIED IN 206 AS RESPONSIBLE FOR CHILD.	As part of this survey, we are asking that children all over the country take a test to see if they have malaria. Malaria is a serious illness caused by a parasite transmitted by a mosquito bite. This survey will help the government to develop programs to prevent malaria.<br>We ask that all children born in 2009 or later take part in malaria testing in this survey and give a few drops of blood from a finger or heel. The equipment used to take the blood is clean and completely safe. It has never been used before and will be thrown away after each test. (We will use blood from the same finger or heel prick made for the anemia test). One blood drop will be tested for malaria immediately, and the result will be told to you right away. A few blood drops will be collected on a slide(s) and taken to a laboratory for testing. You will not be told the results of the laboratory testing. All results will be kept strictly confidential and will not be shared with anyone other than members of our survey team.<br><br>Do you have any questions?<br>You can say yes to the test, or you can say no. It is up to you to decide.<br>Will you allow (NAME OF CHILD) to participate in the malaria testing?		
210	CIRCLE THE APPROPRIATE CODE AND SIGN YOUR NAME.	GRANTED . . . . 1<br>______________ ←<br>(SIGN)<br>REFUSED . . . . 2<br>NOT PRESENT . . . . . 5<br>OTHER 6	GRANTED . . . . 1<br>______________ ←<br>(SIGN)<br>REFUSED . . . . 2<br>NOT PRESENT . . . . . 5<br>OTHER 6	GRANTED . . . . 1<br>______________ ←<br>(SIGN)<br>REFUSED . . . . 2<br>NOT PRESENT . . . . . 5<br>OTHER 6
211	PREPARE EQUIPMENT AND SUPPLIES ONLY FOR THE TEST(S) FOR WHICH CONSENT HAS BEEN OBTAINED AND PROCEED WITH THE TEST(S).			
212	BAR CODE LABEL FOR MALARIA TEST	PUT THE 1ST BAR CODE LABEL HERE.<br>NOT PRESENT 99994<br>REFUSED . . . . . 99995<br>OTHER . . . . 99996<br><br>PUT THE 2ND BAR CODE ON THE RDT, THE 3RD ON THE THICK SMEAR SLIDE, THE 4TH ON THE THIN SMEAR SLIDE AND THE 5TH ON THE TRANSMITTAL FORM.	PUT THE 1ST BAR CODE LABEL HERE.<br>NOT PRESENT 99994<br>REFUSED . . . . . 99995<br>OTHER . . . . 99996<br><br>PUT THE 2ND BAR CODE ON THE RDT, THE 3RD ON THE THICK SMEAR SLIDE, THE 4TH ON THE THIN SMEAR SLIDE AND THE 5TH ON THE TRANSMITTAL FORM.	PUT THE 1ST BAR CODE LABEL HERE.<br>NOT PRESENT 99994<br>REFUSED . . . . . 99995<br>OTHER . . . . 99996<br><br>PUT THE 2ND BAR CODE ON THE RDT, THE 3RD ON THE THICK SMEAR SLIDE, THE 4TH ON THE THIN SMEAR SLIDE AND THE 5TH ON THE TRANSMITTAL FORM.
213	RECORD HEMOGLOBIN LEVEL HERE AND IN THE ANEMIA AND MALARIA BROCHURE.	G/DL [ ][ ] . [ ]<br>NOT PRESENT . . . . . 994<br>REFUSED . . . . 995<br>OTHER 996	G/DL [ ][ ] . [ ]<br>NOT PRESENT . . . . . 994<br>REFUSED . . . . 995<br>OTHER 996	G/DL [ ][ ] . [ ]<br>NOT PRESENT . . . . . 994<br>REFUSED . . . . 995<br>OTHER 996
214	RECORD RESULT CODE OF THE MALARIA RDT.	TESTED . . . . 1<br>NOT PRESENT . . . . . 2<br>REFUSED . . . . 3<br>OTHER . . . . 6<br>(SKIP TO 216)	TESTED . . . . 1<br>NOT PRESENT . . . . . 2<br>REFUSED . . . . 3<br>OTHER . . . . 6<br>(SKIP TO 216)	TESTED . . . . 1<br>NOT PRESENT . . . . . 2<br>REFUSED . . . . 3<br>OTHER . . . . 6<br>(SKIP TO 216)
215	RECORD THE RESULT OF THE MALARIA RDT HERE AND IN THE ANEMIA AND MALARIA BROCHURE.	POSITIVE . . . . 1<br>(SKIP TO 218)<br>NEGATIVE . . . . 2<br>OTHER 6	POSITIVE . . . . 1<br>(SKIP TO 218)<br>NEGATIVE . . . . 2<br>OTHER 6	POSITIVE . . . . 1<br>(SKIP TO 218)<br>NEGATIVE . . . . 2<br>OTHER 6
216	CHECK 213:<br><br>HEMOGLOBIN RESULT	BELOW 8.0 G/DL, SEVERE ANEMIA . . . . 1<br>8.0 G/DL OR ABOVE . . . 2<br>NOT PRESENT . . . . . 4<br>REFUSED . . . . 5<br>OTHER . . . . 6<br>(SKIP TO 229)	BELOW 8.0 G/DL, SEVERE ANEMIA . . . . 1<br>8.0 G/DL OR ABOVE . . . 2<br>NOT PRESENT . . . . . 4<br>REFUSED . . . . 5<br>OTHER . . . . 6<br>(SKIP TO 229)	BELOW 8.0 G/DL, SEVERE ANEMIA . . . . 1<br>8.0 G/DL OR ABOVE . . . 2<br>NOT PRESENT . . . . . 4<br>REFUSED . . . . 5<br>OTHER . . . . 6<br>(SKIP TO 229)

		CHILD 1	CHILD 2	CHILD 3
	NAME FROM COLUMN 2	NAME ________	NAME ________	NAME ________
217	**SEVERE ANEMIA REFERRAL** STATEMENT	The anemia test shows that (NAME OF CHILD) has severe anemia. Your child is very ill and must be taken to a health facility immediately.<br><br>SKIP TO 229		
218	Does (NAME) suffer from the any of following illnesses or symptoms:			
a)	Extreme weakness?	EXTREME WEAKNESS . A	EXTREME WEAKNESS . A	EXTREME WEAKNESS . A
b)	Heart problems?	HEART PROBLEMS . . . B	HEART PROBLEMS . . . B	HEART PROBLEMS . . . B
c)	Loss of consciousness?	LOSS OF CONSCIOUSNESS . . C	LOSS OF CONSCIOUSNESS . . C	LOSS OF CONSCIOUSNESS . . C
d)	Rapid or difficult breathing?	RAPID BREATHING . . . D	RAPID BREATHING . . . D	RAPID BREATHING . . . D
e)	Seizures?	SEIZURES . . . . E	SEIZURES . . . . E	SEIZURES . . . . E
f)	Abnormal bleeding?	BLEEDING . . . . F	BLEEDING . . . . F	BLEEDING . . . . F
g)	Jaundice or yellow skin?	JAUNDICE . . . . G	JAUNDICE . . . . G	JAUNDICE . . . . G
h)	Dark urine?	DARK URINE . . . . H	DARK URINE . . . . H	DARK URINE . . . . H
	IF NONE OF THE ABOVE SYMPTOMS, CIRCLE CODE Y	NONE OF ABOVE SYMPTOMS . . . . . . Y	NONE OF ABOVE SYMPTOMS . . . . . . Y	NONE OF ABOVE SYMPTOMS . . . . . . Y
219	CHECK 218:<br><br>ANY CODE A-H CIRCLED?	ONLY CODE Y CIRCLED . . . . 1<br>ANY CODE A-H CIRCLED . . . . . 2<br>(SKIP TO 222)	ONLY CODE Y CIRCLED . . . . 1<br>ANY CODE A-H CIRCLED . . . . . 2<br>(SKIP TO 222)	ONLY CODE Y CIRCLED . . . . 1<br>ANY CODE A-H CIRCLED . . . . . 2<br>(SKIP TO 222)
220	CHECK 213:<br><br>HEMOGLOBIN RESULT	BELOW 8.0 G/DL, SEVERE ANEMIA . . . . 1<br>(SKIP TO 222)<br>8.0 G/DL OR ABOVE 2<br>NOT PRESENT . . . . . 4<br>REFUSED . . . . 5<br>OTHER 6	BELOW 8.0 G/DL, SEVERE ANEMIA . . . . 1<br>(SKIP TO 222)<br>8.0 G/DL OR ABOVE 2<br>NOT PRESENT . . . . . 4<br>REFUSED . . . . 5<br>OTHER 6	BELOW 8.0 G/DL, SEVERE ANEMIA . . . . 1<br>(SKIP TO 222)<br>8.0 G/DL OR ABOVE 2<br>NOT PRESENT . . . . . 4<br>REFUSED . . . . 5<br>OTHER 6
221	In the past two weeks has (NAME) taken or is taking COARTEM/ACT given by a doctor or health center to treat the malaria?<br><br>VERIFY BY ASKING TO SEE TREATMENT.	YES . . . . 1<br>(SKIP TO 223)<br>NO . . . . 2<br>(SKIP TO 223A)	YES . . . . 1<br>(SKIP TO 223)<br>NO . . . . 2<br>(SKIP TO 223A)	YES . . . . 1<br>(SKIP TO 223)<br>NO . . . . 2<br>(SKIP TO 223A)
222	**SEVERE MALARIA REFERRAL** STATEMENT	The malaria test shows that (NAME OF CHILD) has malaria. Your child also has symptoms of severe malaria. The malaria treatment I have will not help your child, and I cannot give you the medication. Your child is very ill and must be taken to a health facility right away.<br><br>SKIP TO 228		
223	**ALREADY TAKING [FIRST LINE MEDICATION] REFERRAL STATEMENT**	You have told me that (NAME OF CHILD) has already received COARTEM/ACT for malaria. Therefore, I cannot give you additional COARTEM/ACT. However, the test shows that he/she has malaria. If your child has a fever for two days after the last dose of COARTEM/ACT, you should take the child to the nearest health facility for further examination.<br><br>SKIP TO 228		

<table>
<tr><td></td><td></td><td>CHILD 1</td><td>CHILD 2</td><td>CHILD 3</td></tr>
<tr><td></td><td>NAME FROM COLUMN 2</td><td>NAME ________________</td><td>NAME ________________</td><td>NAME ________________</td></tr>
<tr><td>223A</td><td>CHECK 203:
IS CHILD AGE 0-3 MONTHS, I.E., WAS CHILD BORN IN MONTH OF INTERVIEW OR THREE PREVIOUS MONTHS?</td><td>0-3 MONTHS 1
OLDER 2
SKIP TO 224</td><td>0-3 MONTHS 1
OLDER 2
SKIP TO 224</td><td>0-3 MONTHS 1
OLDER 2
SKIP TO 224</td></tr>
<tr><td>223B</td><td><u>UNDER 4 MONTHS MALARIA REFERRAL STATEMENT</u></td><td colspan="3">The malaria test shows that (NAME OF CHILD) has malaria. Your child is also younger than 4 months old and therefore requires special treatment from a health facility. Your child is ill and must be taken to a health facility right away.

SKIP TO 228</td></tr>
<tr><td>224</td><td>READ INFORMATION FOR MALARIA TREATMENT AND CONSENT STATEMENT TO PARENT OR OTHER ADULT RESPONSIBLE FOR THE CHILD.</td><td colspan="3">The malaria test shows that your child has malaria. We can give you free medicine. The medicine is called COARTEM/ACT. COARTEM/ACT is very effective and in a few days it should get rid of the fever and other symptoms. You do not have to give the child the medicine. This is up to you. Please tell me whether you accept the medicine or not.</td></tr>
<tr><td>225</td><td>CIRCLE THE APPROPRIATE CODE AND SIGN YOUR NAME.</td><td>ACCEPTED MEDICINE . . 1
____
(SIGN)
REFUSED 2
OTHER . . . . 6</td><td>ACCEPTED MEDICINE . . 1
____
(SIGN)
REFUSED 2
OTHER . . . . 6</td><td>ACCEPTED MEDICINE . . 1
____
(SIGN)
REFUSED 2
OTHER . . . . 6</td></tr>
<tr><td>226</td><td>CHECK 225:

MEDICATION ACCEPTED</td><td>ACCEPTED MEDICINE . . 1
REFUSED 2
OTHER 6
(SKIP TO 228)</td><td>ACCEPTED MEDICINE . . 1
REFUSED 2
OTHER 6
(SKIP TO 228)</td><td>ACCEPTED MEDICINE . . 1
REFUSED 2
OTHER 6
(SKIP TO 228)</td></tr>
<tr><td>227</td><td>TREATMENT FOR CHILDREN WITH POSITIVE MALARIA TESTS</td><td colspan="3">TREATMENT WITH COARTEM/ACT

Weight (in Kg) – Approximate age | Dosage *
Under 4 months | Refer to health facility
5 kgs. to 14 kgs. (from 4 months up to 3 years) | 1 tablet twice daily for 3 days
15 kgs. to 24 kgs. (from 3 years up to 7 years) | 2 tablets twice daily for 3 days
* Co-formulated tablets containing 20 mg Artemether and 120 mg Lumefantrine per tablet

First day starts by taking first dose followed by the second one 8 hours later; on subsequent days the recommendation is simply “morning” and “evening” (usually around 12 hours apart).Take the medicine (crushed for smaller children) with high fat food or drinks like milk.

Make sure that the FULL 3 days treatment is taken at the recommended times, otherwise the infection may return. If your child vomits within an hour of taking the medicine, you will need to get additional tablets and repeat the dose.

ALSO TELL THE PARENT/ADULT RESPONSIBLE FOR THE CHILD: If [NAME] has a high fever, fast or difficult breathing, is not able to drink or breastfeed, gets sicker or does not get better in two days, you should take him/her to a health professional for treatment right away.</td></tr>
<tr><td>228</td><td>RECORD THE RESULT CODE OF <u>MALARIA TREATMENT OR REFERRAL.</u></td><td>MEDICATION GIVEN . . . 1
MEDS REFUSED 2
SEVERE MALARIA
REFERRAL 3
ALREADY TAKING ACTS
REFERRAL 4
UNDER 4 MONTHS
REFERRAL 5
OTHER . . . . 6</td><td>MEDICATION GIVEN . . . 1
MEDS REFUSED 2
SEVERE MALARIA
REFERRAL 3
ALREADY TAKING ACTS
REFERRAL 4
UNDER 4 MONTHS
REFERRAL 5
OTHER . . . . 6</td><td>MEDICATION GIVEN . . . 1
MEDS REFUSED 2
SEVERE MALARIA
REFERRAL 3
ALREADY TAKING ACTS
REFERRAL 4
UNDER 4 MONTHS
REFERRAL 5
OTHER . . . . 6</td></tr>
<tr><td>229</td><td colspan="4">GO BACK TO 202 IN NEXT COLUMN OF THIS QUESTIONNAIRE OR IN THE FIRST COLUMN OF THE NEXT PAGE; IF NO MORE CHILDREN, END INTERVIEW.</td></tr>
</table>

HEMOGLOBIN MEASUREMENT AND MALARIA TESTING FOR CHILDREN AGE 0-5				
201	CHECK COLUMN 9 IN HOUSEHOLD SCHEDULE. RECORD THE LINE NUMBER AND NAME FOR ALL ELIGIBLE CHILDREN 0-5 YEARS IN QUESTION 202. IF MORE THAN SIX CHILDREN, USE ADDITIONAL QUESTIONNAIRE(S).			
		CHILD 4	CHILD 5	CHILD 6
202	LINE NUMBER FROM COLUMN 9<br><br>NAME FROM COLUMN 2	LINE NUMBER . . . . .<br><br>NAME ________	LINE NUMBER . . . . .<br><br>NAME ________	LINE NUMBER . . . . .<br><br>NAME ________
203	IF MOTHER INTERVIEWED, COPY MONTH AND YEAR OF BIRTH FROM BIRTH HISTORY AND ASK DAY; IF MOTHER NOT INTERVIEWED, ASK:<br>What is (NAME'S) birth date?	DAY . . . .<br>MONTH . . . . .<br>YEAR	DAY . . . .<br>MONTH . . . . .<br>YEAR	DAY . . . .<br>MONTH . . . . .<br>YEAR
204	CHECK 203:<br>CHILD BORN IN JANUARY 2009 OR LATER?	YES . . . . 1<br>NO . . . . 2<br>(GO TO 203 FOR NEXT CHILD OR, IF NO MORE CHILDREN, END INTERVIEW)	YES . . . . 1<br>NO . . . . 2<br>(GO TO 203 FOR NEXT CHILD OR, IF NO MORE CHILDREN, END INTERVIEW)	YES . . . . 1<br>NO . . . . 2<br>(GO TO 203 FOR NEXT CHILD OR, IF NO MORE CHILDREN, END INTERVIEW)
206	LINE NUMBER OF PARENT/ OTHER ADULT RESPONSIBLE FOR THE CHILD (FROM COLUMN 1 OF HOUSEHOLD SCHEDULE). RECORD '00' IF NOT LISTED.	LINE NUMBER	LINE NUMBER	LINE NUMBER . . . . .
207	ASK CONSENT FOR ANEMIA TEST FROM PARENT/OTHER ADULT IDENTIFIED IN 206 AS RESPONSIBLE FOR CHILD.	As part of this survey, we are asking children all over the country to take an anemia test. Anemia is a serious health problem that usually results from poor nutrition, infection, or chronic disease. This survey will assist the government to develop programs to prevent and treat anemia.<br>We ask that all children born in 2009 or later take part in anemia testing in this survey and give a few drops of blood from a finger or heel. The equipment used to take the blood is clean and completely safe. It has never been used before and will be thrown away after each test.<br>The blood will be tested for anemia immediately, and the result will be told to you right away. The result will be kept strictly confidential and will not be shared with anyone other than members of our survey team.<br><br>Do you have any questions?<br>You can say yes to the test, or you can say no. It is up to you to decide.<br>Will you allow (NAME OF CHILD) to participate in the anemia test?		
208	CIRCLE THE APPROPRIATE CODE AND SIGN YOUR NAME.	GRANTED . . . . 1<br>________<br>(SIGN)<br>REFUSED . . . . 2<br>NOT PRESENT . . . . . 5<br>OTHER 6	GRANTED . . . . 1<br>________<br>(SIGN)<br>REFUSED . . . . 2<br>NOT PRESENT . . . . . 5<br>OTHER 6	GRANTED . . . . 1<br>________<br>(SIGN)<br>REFUSED . . . . 2<br>NOT PRESENT . . . . . 5<br>OTHER 6

		CHILD 4	CHILD 5	CHILD 6
	NAME FROM COLUMN 2	NAME ________	NAME ________	NAME ________
209	ASK CONSENT FOR MALARIA TEST FROM PARENT/OTHER ADULT IDENTIFIED IN 206 AS RESPONSIBLE FOR CHILD.	As part of this survey, we are asking that children all over the country take a test to see if they have malaria. Malaria is a serious illness caused by a parasite transmitted by a mosquito bite. This survey will help the government to develop programs to prevent malaria.<br>We ask that all children born in 2009 or later take part in malaria testing in this survey and give a few drops of blood from a finger or heel. The equipment used to take the blood is clean and completely safe. It has never been used before and will be thrown away after each test. (We will use blood from the same finger or heel prick made for the anemia test). One blood drop will be tested for malaria immediately, and the result will be told to you right away. A few blood drops will be collected on a slide(s) and taken to a laboratory for testing. You will not be told the results of the laboratory testing. All results will be kept strictly confidential and will not be shared with anyone other than members of our survey team.<br><br>Do you have any questions?<br>You can say yes to the test, or you can say no. It is up to you to decide.<br>Will you allow (NAME OF CHILD) to participate in the malaria testing?		
210	CIRCLE THE APPROPRIATE CODE AND SIGN YOUR NAME.	GRANTED . . . . 1<br>________ (SIGN)<br>REFUSED . . . . 2<br>NOT PRESENT . . . . . 5<br>OTHER 6	GRANTED . . . . 1<br>________ (SIGN)<br>REFUSED . . . . 2<br>NOT PRESENT . . . . . 5<br>OTHER 6	GRANTED . . . . 1<br>________ (SIGN)<br>REFUSED . . . . 2<br>NOT PRESENT . . . . . 5<br>OTHER 6
211	PREPARE EQUIPMENT AND SUPPLIES ONLY FOR THE TEST(S) FOR WHICH CONSENT HAS BEEN OBTAINED AND PROCEED WITH THE TEST(S).			
212	BAR CODE LABEL FOR MALARIA TEST	PUT THE 1ST BAR CODE LABEL HERE.<br>NOT PRESENT 99994<br>REFUSED . . . . . 99995<br>OTHER . . . . 99996<br><br>PUT THE 2ND BAR CODE ON THE RDT, THE 3RD ON THE THICK SMEAR SLIDE, THE 4TH ON THE THIN SMEAR SLIDE AND THE 5TH ON THE TRANSMITTAL FORM.	PUT THE 1ST BAR CODE LABEL HERE.<br>NOT PRESENT 99994<br>REFUSED . . . . . 99995<br>OTHER . . . . 99996<br><br>PUT THE 2ND BAR CODE ON THE RDT, THE 3RD ON THE THICK SMEAR SLIDE, THE 4TH ON THE THIN SMEAR SLIDE AND THE 5TH ON THE TRANSMITTAL FORM.	PUT THE 1ST BAR CODE LABEL HERE.<br>NOT PRESENT 99994<br>REFUSED . . . . . 99995<br>OTHER . . . . 99996<br><br>PUT THE 2ND BAR CODE ON THE RDT, THE 3RD ON THE THICK SMEAR SLIDE, THE 4TH ON THE THIN SMEAR SLIDE AND THE 5TH ON THE TRANSMITTAL FORM.
213	RECORD HEMOGLOBIN LEVEL HERE AND IN THE ANEMIA AND MALARIA BROCHURE.	G/DL [ ][ ].[ ]<br>NOT PRESENT . . . . .994<br>REFUSED . . . . .995<br>OTHER 996	G/DL [ ][ ].[ ]<br>NOT PRESENT . . . . .994<br>REFUSED . . . . .995<br>OTHER 996	G/DL [ ][ ].[ ]<br>NOT PRESENT . . . . .994<br>REFUSED . . . . .995<br>OTHER 996
214	RECORD RESULT CODE OF THE MALARIA RDT.	TESTED . . . . 1<br>NOT PRESENT . . . . . 2<br>REFUSED . . . . 3<br>OTHER . . . . 6<br>(SKIP TO 216)	TESTED . . . . 1<br>NOT PRESENT . . . . . 2<br>REFUSED . . . . 3<br>OTHER . . . . 6<br>(SKIP TO 216)	TESTED . . . . 1<br>NOT PRESENT . . . . . 2<br>REFUSED . . . . 3<br>OTHER . . . . 6<br>(SKIP TO 216)
215	RECORD THE RESULT OF THE MALARIA RDT HERE AND IN THE ANEMIA AND MALARIA BROCHURE.	POSITIVE . . . . 1<br>(SKIP TO 218)<br>NEGATIVE . . . . 2<br>OTHER 6	POSITIVE . . . . 1<br>(SKIP TO 218)<br>NEGATIVE . . . . 2<br>OTHER 6	POSITIVE . . . . 1<br>(SKIP TO 218)<br>NEGATIVE . . . . 2<br>OTHER 6
216	CHECK 213:<br><br>HEMOGLOBIN RESULT	BELOW 8.0 G/DL, SEVERE ANEMIA . . . . 1<br>8.0 G/DL OR ABOVE . . . 2<br>NOT PRESENT . . . . . 4<br>REFUSED . . . . 5<br>OTHER . . . . 6<br>(SKIP TO 229)	BELOW 8.0 G/DL, SEVERE ANEMIA . . . . 1<br>8.0 G/DL OR ABOVE . . . 2<br>NOT PRESENT . . . . . 4<br>REFUSED . . . . 5<br>OTHER . . . . 6<br>(SKIP TO 229)	BELOW 8.0 G/DL, SEVERE ANEMIA . . . . 1<br>8.0 G/DL OR ABOVE . . . 2<br>NOT PRESENT . . . . . 4<br>REFUSED . . . . 5<br>OTHER . . . . 6<br>(SKIP TO 229)

		CHILD 4	CHILD 5	CHILD 6
	NAME FROM COLUMN 2	NAME ________________	NAME ________________	NAME ________________
217	**SEVERE ANEMIA REFERRAL** STATEMENT	The anemia test shows that (NAME OF CHILD) has severe anemia. Your child is very ill and must be taken to a health facility immediately.<br><br>SKIP TO 229		
218	Does (NAME) suffer from the any of following illnesses or symptoms:			
a)	Extreme weakness?	EXTREME WEAKNESS . A	EXTREME WEAKNESS . A	EXTREME WEAKNESS . A
b)	Heart problems?	HEART PROBLEMS . . . B	HEART PROBLEMS . . . B	HEART PROBLEMS . . . B
c)	Loss of consciousness?	LOSS OF CONSCIOUSNESS . . . C	LOSS OF CONSCIOUSNESS . . . C	LOSS OF CONSCIOUSNESS . . . C
d)	Rapid or difficult breathing?	RAPID BREATHING . . . D	RAPID BREATHING . . . D	RAPID BREATHING . . . D
e)	Seizures?	SEIZURES . . . . E	SEIZURES . . . . E	SEIZURES . . . . E
f)	Abnormal bleeding?	BLEEDING . . . . F	BLEEDING . . . . F	BLEEDING . . . . F
g)	Jaundice or yellow skin?	JAUNDICE . . . . G	JAUNDICE . . . . G	JAUNDICE . . . . G
h)	Dark urine?	DARK URINE . . . . H	DARK URINE . . . . H	DARK URINE . . . . H
	IF NONE OF THE ABOVE SYMPTOMS, CIRCLE CODE Y	NONE OF ABOVE SYMPTOMS . . . . . . Y	NONE OF ABOVE SYMPTOMS . . . . . . Y	NONE OF ABOVE SYMPTOMS . . . . . . Y
219	CHECK 218:<br><br>ANY CODE A-H CIRCLED?	ONLY CODE Y CIRCLED . . . . 1<br>ANY CODE A-H CIRCLED . . . . . 2<br>(SKIP TO 222)	ONLY CODE Y CIRCLED . . . . 1<br>ANY CODE A-H CIRCLED . . . . . 2<br>(SKIP TO 222)	ONLY CODE Y CIRCLED . . . . 1<br>ANY CODE A-H CIRCLED . . . . . 2<br>(SKIP TO 222)
220	CHECK 213:<br><br>HEMOGLOBIN RESULT	BELOW 8.0 G/DL, SEVERE ANEMIA . . . . 1<br>(SKIP TO 222)<br>8.0 G/DL OR ABOVE . . . 2<br>NOT PRESENT . . . . . 4<br>REFUSED . . . . 5<br>OTHER 6	BELOW 8.0 G/DL, SEVERE ANEMIA . . . . 1<br>(SKIP TO 222)<br>8.0 G/DL OR ABOVE . . . 2<br>NOT PRESENT . . . . . 4<br>REFUSED . . . . 5<br>OTHER 6	BELOW 8.0 G/DL, SEVERE ANEMIA . . . . 1<br>(SKIP TO 222)<br>8.0 G/DL OR ABOVE . . . 2<br>NOT PRESENT . . . . . 4<br>REFUSED . . . . 5<br>OTHER 6
221	In the past two weeks has (NAME) taken or is taking COARTEM/ACT given by a doctor or health center to treat the malaria?<br><br>VERIFY BY ASKING TO SEE TREATMENT.	YES . . . . 1<br>(SKIP TO 223)<br><br>NO . . . . 2<br>(SKIP TO 223A)	YES . . . . 1<br>(SKIP TO 223)<br><br>NO . . . . 2<br>(SKIP TO 223A)	YES . . . . 1<br>(SKIP TO 223)<br><br>NO . . . . 2<br>(SKIP TO 223A)
222	**SEVERE MALARIA REFERRAL** STATEMENT	The malaria test shows that (NAME OF CHILD) has malaria. Your child also has symptoms of severe malaria. The malaria treatment I have will not help your child, and I cannot give you the medication. Your child is very ill and must be taken to a health facility right away.<br><br>SKIP TO 228		
223	**ALREADY TAKING [FIRST LINE MEDICATION] REFERRAL STATEMENT**	You have told me that (NAME OF CHILD) has already received COARTEM/ACT for malaria. Therefore, I cannot give you additional COARTEM/ACT. However, the test shows that he/she has malaria. If your child has a fever for two days after the last dose of COARTEM/ACT, you should take the child to the nearest health facility for further examination.<br><br>SKIP TO 228		

<table>
<tr><td></td><td></td><td>CHILD 4</td><td>CHILD 5</td><td>CHILD 6</td></tr>
<tr><td></td><td>NAME FROM COLUMN 2</td><td>NAME ________________</td><td>NAME ________________</td><td>NAME ________________</td></tr>
<tr><td>223A</td><td>CHECK 203:
IS CHILD AGE 0-3 MONTHS, I.E., WAS CHILD BORN IN MONTH OF INTERVIEW OR THREE PREVIOUS MONTHS?</td><td>0-3 MONTHS 1
OLDER 2
SKIP TO 224</td><td>0-3 MONTHS 1
OLDER 2
SKIP TO 224</td><td>0-3 MONTHS 1
OLDER 2
SKIP TO 224</td></tr>
<tr><td>223B</td><td><u>UNDER 4 MONTHS MALARIA REFERRAL STATEMENT</u></td><td colspan="3">The malaria test shows that (NAME OF CHILD) has malaria. Your child is also younger than 4 months old and therefore requires special treatment from a health facility. Your child is ill and must be taken to a health facility right away.

SKIP TO 228</td></tr>
<tr><td>224</td><td>READ INFORMATION FOR MALARIA TREATMENT AND CONSENT STATEMENT TO PARENT OR OTHER ADULT RESPONSIBLE FOR THE CHILD.</td><td colspan="3">The malaria test shows that your child has malaria. We can give you free medicine. The medicine is called COARTEM/ACT. COARTEM/ACT is very effective and in a few days it should get rid of the fever and other symptoms. You do not have to give the child the medicine. This is up to you. Please tell me whether you accept the medicine or not.</td></tr>
<tr><td>225</td><td>CIRCLE THE APPROPRIATE CODE AND SIGN YOUR NAME.</td><td>ACCEPTED MEDICINE . . 1
____
(SIGN)
REFUSED 2
OTHER . . . . 6</td><td>ACCEPTED MEDICINE . . 1
____
(SIGN)
REFUSED 2
OTHER . . . . 6</td><td>ACCEPTED MEDICINE . . 1
____
(SIGN)
REFUSED 2
OTHER . . . . 6</td></tr>
<tr><td>226</td><td>CHECK 225:

MEDICATION ACCEPTED</td><td>ACCEPTED MEDICINE . . 1
REFUSED 2
OTHER 6
(SKIP TO 228)</td><td>ACCEPTED MEDICINE . . 1
REFUSED 2
OTHER 6
(SKIP TO 228)</td><td>ACCEPTED MEDICINE . . 1
REFUSED 2
OTHER 6
(SKIP TO 228)</td></tr>
<tr><td>227</td><td>TREATMENT FOR CHILDREN WITH POSITIVE MALARIA TESTS</td><td colspan="3">TREATMENT WITH COARTEM/ACT

Weight (in Kg) – Approximate age | Dosage *
Under 4 months | Refer to health facility
5 kgs. to 14 kgs. (from 4 month up to 3 years) | 1 tablet twice daily for 3 days
15 kgs. to 24 kgs. (from 3 years up to 7 years) | 2 tablets twice daily for 3 days
* Co-formulated tablets containing 20 mg Artemether and 120 mg Lumefantrine per tablet

First day starts by taking first dose followed by the second one 8 hours later; on subsequent days the recommendation is simply “morning” and “evening” (usually around 12 hours apart).Take the medicine (crushed for smaller children) with high fat food or drinks like milk.

Make sure that the FULL 3 days treatment is taken at the recommended times, otherwise the infection may return. If your child vomits within an hour of taking the medicine, you will need to get additional tablets and repeat the dose.

ALSO TELL THE PARENT/ADULT RESPONSIBLE FOR THE CHILD: If [NAME] has a high fever, fast or difficult breathing, is not able to drink or breastfeed, gets sicker or does not get better in two days, you should take him/her to a health professional for treatment right away.</td></tr>
<tr><td>228</td><td>RECORD THE RESULT CODE OF <u>MALARIA TREATMENT OR REFERRAL.</u></td><td>MEDICATION GIVEN . . . 1
MEDS REFUSED 2
SEVERE MALARIA
REFERRAL 3
ALREADY TAKING ACTS
REFERRAL 4
UNDER 4 MONTHS
REFERRAL 5
OTHER . . . . 6</td><td>MEDICATION GIVEN . . . 1
MEDS REFUSED 2
SEVERE MALARIA
REFERRAL 3
ALREADY TAKING ACTS
REFERRAL 4
UNDER 4 MONTHS
REFERRAL 5
OTHER . . . . 6</td><td>MEDICATION GIVEN . . . 1
MEDS REFUSED 2
SEVERE MALARIA
REFERRAL 3
ALREADY TAKING ACTS
REFERRAL 4
UNDER 4 MONTHS
REFERRAL 5
OTHER . . . . 6</td></tr>
<tr><td>229</td><td colspan="4">GO BACK TO 202 IN NEXT COLUMN OF THIS QUESTIONNAIRE OR IN THE FIRST COLUMN OF THE NEXT PAGE; IF NO MORE CHILDREN, END INTERVIEW.</td></tr>
</table>
