# Supplementary material for: Long lasting insecticidal bed nets ownership, access and use in a high malaria transmission setting before and after a mass distribution campaign in Uganda
Source: PLoS One. 2018 Jan 18;13(1):e0191191. doi: 10.1371/journal.pone.0191191 (PMC5773208; doi:10.1371/journal.pone.0191191)
Supplement: S2 File — (PDF) [file pone.0191191.s002.pdf]

2014 UGANDA MALARIA INDICATOR SURVEY
WOMAN'S QUESTIONNAIRE

UGANDA
IDRC/MOH/UBOS

FORMATTING DATE: 19 November 2014
LANGUAGE DATE: 19 November 2014

IDENTIFICATION (1)

REGION ______________________
DISTRICT ______________________
COUNTY ______________________
SUBCOUNTY/TOWN ______________________
PARISH/LC2 NAME ______________________
EA NAME ______________________
UMIS NUMBER . . . .
URBAN=1, PERI URBAN=2, RURAL=3 . . . .
NAME AND LINE NUMBER OF WOMAN ______________________
HOUSEHOLD NUMBER . . . .
HOUSEHOLD SAMPLE NUMBER . . . .

INTERVIEWER VISITS

	1	2	3	FINAL VISIT
DATE	________	________	________	DAY<br>MONTH<br>YEAR
INTERVIEWER'S NAME	________	________	________	INT. NUMBER
RESULT*	________	________	________	RESULT
NEXT VISIT: DATE	________	________		TOTAL NUMBER OF VISITS
TIME	________	________		

*RESULT CODES:
1 COMPLETED
2 NOT AT HOME
3 POSTPONED
4 REFUSED
5 PARTLY COMPLETED
6 INCAPACITATED
7 OTHER ______________________ (SPECIFY)

LANGUAGE OF THE QUESTIONNAIRE 7
LANGUAGE USED IN THE INTERVIEW . . . .
NATIVE LANGUAGE OF RESPONDENT . . . .
TRANSLATOR USED (NOT AT ALL=1; SOMETIMES=2; ALL THE TIME=3) . . . .
LANGUAGE OF THE QUESTIONNAIRE **English**

LANGUAGE USED:
1 ATESO-KARAMOJONG
2 LUGANDA
3 LUGBARA
4 LUO
5 RUNYANKORE-RUKIGA
6 RUNYORO-RUTORO
7 ENGLISH
8 OTHER ______________ (SPECIFY)

SUPERVISOR	OFFICE EDITOR	KEYED BY
NAME ______________		

SECTION 1. RESPONDENT'S BACKGROUND

INTRODUCTION AND CONSENT

INFORMED CONSENT

Hello. My name is ______________________________________. I am working with MOH. We are conducting a survey about malaria all over UGANDA The information we collect will help the government to plan health services. Your household was selected for the survey. The questions usually take about 10-20 minutes. All of the answers you give will be confidential and will not be shared with anyone other than members of our survey team. You don't have to be in the survey, but we hope you will agree to answer the questions since your views are important. If I ask you any question you don't want to answer, just let me know and I will go on to the next question or you can stop the interview at any time.

In case you need more information about the survey, you may contact the person listed on the card that has already been given to your household.
Do you have any questions? May I begin the interview now?

SIGNATURE OF INTERVIEWER: ______________________ DATE: ______________

RESPONDENT AGREES TO BE INTERVIEWED . . . 1 ↓ RESPONDENT DOES NOT AGREE TO BE INTERVIEWED . . . 2 → END

NO.	QUESTIONS AND FILTERS	CODING CATEGORIES	SKIP
101	RECORD THE TIME.	HOUR . . . . [ ][ ]<br>MINUTES . . . . [ ][ ]	
102	In what month and year were you born?	MONTH . . . . [ ][ ]<br>DON'T KNOW MONTH . . . . 98<br>YEAR . . . . [ ][ ][ ][ ]<br>DON'T KNOW YEAR 9998	
103	How old were you at your last birthday?<br><br>COMPARE AND CORRECT 102 AND/OR 103 IF INCONSISTENT.	AGE IN COMPLETED YEARS [ ][ ]	
104	Have you ever attended school?	YES . . . . 1<br>NO 2	 → 108
105	What is the highest level of school you attended: primary, 'O' level 'A' level or university or tertiary ?	PRIMARY . . . . 1<br>'O' LEVEL . . . . 2<br>'A' LEVEL . . . . 3<br>UNIVERSITY/TERTIARY 4	
106	What is the highest (class/year) you completed at that level?<br><br>IF COMPLETED LESS THAN ONE YEAR AT THAT LEVEL, RECORD '00'.	CLASS/YEAR . . . . [ ][ ]	
107	CHECK 105:<br>PRIMARY [ ] ↓ SECONDARY OR HIGHER [ ]		→ 109

NO.	QUESTIONS AND FILTERS	CODING CATEGORIES	SKIP
108	Now I would like you to read this sentence to me.<br><br>SHOW CARD TO RESPONDENT.<br><br>IF RESPONDENT CANNOT READ WHOLE SENTENCE, PROBE:<br><br>Can you read any part of the sentence to me?	CANNOT READ AT ALL . . . . 1<br>ABLE TO READ ONLY PARTS OF SENTENCE . . . . 2<br>ABLE TO READ WHOLE SENTENCE . . . 3<br>NO CARD WITH REQUIRED LANGUAGE ______ 4<br>(SPECIFY LANGUAGE)<br>BLIND/VISUALLY IMPAIRED 5	
109	What is your religion?	CATHOLIC . . . . 01<br>ANGLICAN/PROTESTANT . . . . 02<br>SDA . . . . 03<br>ORTHODOX . . . . 04<br>PENTECOSTAL . . . . 05<br>OTHER CHRISTIAN . . . . 06<br>MOSLEM . . . . 07<br>BAHAI . . . . 08<br>TRADITIONAL . . . . 09<br>HINDU . . . . 10<br>NONE . . . . 11<br>OTHER ______ 96<br>(SPECIFY)	
110	What is your ethnic group?	BAGANDA . . . . 01<br>BANYANKORE . . . . 02<br>ITESO . . . . 03<br>LUGBARA/MADI . . . . 04<br>BASOGA . . . . 05<br>LANGI . . . . 06<br>BAKIGA . . . . 07<br>KARIMOJONG . . . . 08<br>ACHOLI . . . . 09<br>BAGISU/SABINY . . . . 10<br>ALUR/JOPADHOLA . . . . 11<br>BANYORO . . . . 12<br>BATORO . . . . 13<br>OTHER ______ 96<br>(SPECIFY)	
111	In the past six months, have you seen or heard any messages about malaria?	YES . . . . 1<br>NO 2	 → 201
112	Have you seen or heard these messages:<br>a) On the radio?<br>b) On the television?<br>c) On a poster or billboard?<br>d) From a community health worker?<br><br>e) At a community event?<br>f) Anywhere else?	**YES** **NO**<br>a) RADIO . . . . 1 2<br>b) TELEVISION . . . . 1 2<br>c) POSTER OR BILLBOARD . . 1 2<br>d) COMMUNITY HEALTH WORKER . . . . 1 2<br>e) COMMUNITY EVENT . . . . . 1 2<br>f) ANYWHERE ELSE 1 2	

SECTION 2. REPRODUCTION

NO.	QUESTIONS AND FILTERS	CODING CATEGORIES	SKIP
201	Now I would like to ask about all the births you have had during your life. Have you ever given birth?	YES . . . . 1<br>NO 2	 → 206
202	Do you have any sons or daughters to whom you have given birth who are now living with you?	YES . . . . 1<br>NO 2	 → 204
203	a) How many sons live with you?<br>b) And how many daughters live with you?<br>IF NONE, RECORD '00'.	a) SONS AT HOME . . . . . [ ][ ]<br>b) DAUGHTERS AT HOME . . . . . [ ][ ]	
204	Do you have any sons or daughters to whom you have given birth who are alive but do not live with you?	YES . . . . 1<br>NO 2	 → 206
205	a) How many sons are alive but do not live with you?<br>b) And how many daughters are alive but do not live with you?<br>IF NONE, RECORD '00'.	a) SONS ELSEWHERE . . . . [ ][ ]<br>b) DAUGHTERS ELSEWHERE . . . [ ][ ]	
206	Have you ever given birth to a boy or girl who was born alive but later died?<br>IF NO, PROBE:<br>Any baby who cried or showed signs of life but did not survive?	YES . . . . 1<br>NO 2	 → 208
207	a) How many boys have died?<br>b) And how many girls have died?<br>IF NONE, RECORD '00'.	a) BOYS DEAD . . . . [ ][ ]<br>b) GIRLS DEAD . . . . [ ][ ]	
208	SUM ANSWERS TO 203, 205, AND 207, AND ENTER TOTAL.<br>IF NONE, RECORD '00'.	TOTAL BIRTHS . . . . [ ][ ]	
209	CHECK 208:<br>Just to make sure that I have this right: you have had in TOTAL _____ births during your life. Is that correct?<br>YES [ ] ↓ NO [ ] → PROBE AND CORRECT 201-208 AS NECESSARY.		
210	Now I'd like to ask you about your more recent births. How many births have you had in the last 6 years?<br>IF NONE, CIRCLE '00.'	TOTAL IN THE LAST 6 YEARS . . . . . . [ ][ ]<br>NONE 00	 → 224

211	Now I would like to record the names of all your births in the last six years, whether still alive or not, starting with the most recent one you had.<br>RECORD NAMES OF ALL THE BIRTHS IN THE LAST 6 YEARS IN 212. RECORD TWINS AND TRIPLETS ON SEPARATE ROWS.							
212<br><br>What name was given to your (most recent/previous) baby?<br>RECORD NAME.<br><br>BIRTH HISTORY NUMBER	213<br><br>Is (NAME) a boy or a girl?	214<br><br>Were any of these births twins?	215<br><br>In what month and year was (NAME) born?<br><br>PROBE: When is his/her birthday?	216<br><br>Is (NAME) still alive?	217<br>IF ALIVE:<br><br>How old was (NAME) at his/her last birthday?<br><br>RECORD AGE IN COMPLETED YEARS.	218<br>IF ALIVE:<br><br>Is (NAME) living with you?	219<br>IF ALIVE:<br><br>RECORD HOUSE-HOLD LINE NUMBER OF CHILD (RECORD '00' IF CHILD NOT LISTED IN HOUSE-HOLD).	220<br><br>Were there any other live births between (NAME) and (NAME OF PREVIOUS BIRTH), including any children who died after birth?
01	BOY 1<br>GIRL 2	SING 1<br>MULT 2	MONTH<br>YEAR	YES . . . . 1<br>NO . . . . 2<br>(NEXT BIRTH)	AGE IN YEARS	YES . . . 1<br>NO 2	HOUSEHOLD LINE NUMBER<br>(NEXT BIRTH)	
02	BOY 1<br>GIRL 2	SING 1<br>MULT 2	MONTH<br>YEAR	YES . . . . 1<br>NO . . . . 2<br>220	AGE IN YEARS	YES . . . 1<br>NO 2	HOUSEHOLD LINE NUMBER	YES . . . . . . 1<br>ADD BIRTH<br>NO . . . . 2<br>NEXT BIRTH
03	BOY 1<br>GIRL 2	SING 1<br>MULT 2	MONTH<br>YEAR	YES . . . . 1<br>NO . . . . 2<br>220	AGE IN YEARS	YES . . . 1<br>NO 2	HOUSEHOLD LINE NUMBER	YES . . . . . . 1<br>ADD BIRTH<br>NO . . . . 2<br>NEXT BIRTH
04	BOY 1<br>GIRL 2	SING 1<br>MULT 2	MONTH<br>YEAR	YES . . . . 1<br>NO . . . . 2<br>220	AGE IN YEARS	YES . . . 1<br>NO 2	HOUSEHOLD LINE NUMBER	YES . . . . . . 1<br>ADD BIRTH<br>NO . . . . 2<br>NEXT BIRTH
05	BOY 1<br>GIRL 2	SING 1<br>MULT 2	MONTH<br>YEAR	YES . . . . 1<br>NO . . . . 2<br>220	AGE IN YEARS	YES . . . 1<br>NO 2	HOUSEHOLD LINE NUMBER	YES . . . . . . 1<br>ADD BIRTH<br>NO . . . . 2<br>NEXT BIRTH
06	BOY 1<br>GIRL 2	SING 1<br>MULT 2	MONTH<br>YEAR	YES . . . . 1<br>NO . . . . 2<br>220	AGE IN YEARS	YES . . . 1<br>NO 2	HOUSEHOLD LINE NUMBER	YES . . . . . . 1<br>ADD BIRTH<br>NO . . . . 2<br>NEXT BIRTH
07	BOY 1<br>GIRL 2	SING 1<br>MULT 2	MONTH<br>YEAR	YES . . . . 1<br>NO . . . . 2<br>220	AGE IN YEARS	YES . . . 1<br>NO 2	HOUSEHOLD LINE NUMBER	YES . . . . . . 1<br>ADD BIRTH<br>NO 2

NO.	QUESTIONS AND FILTERS	CODING CATEGORIES	SKIP
221	Have you had any live births since the birth of (NAME OF MOST RECENT BIRTH)? IF YES, RECORD BIRTH(S) IN TABLE.	YES . . . . 1<br>NO 2	
222	COMPARE 210 WITH NUMBER OF BIRTHS IN HISTORY ABOVE AND MARK:<br>NUMBERS ARE SAME ☐<br>NUMBERS ARE DIFFERENT ☐ → (PROBE AND RECONCILE.)		
223	CHECK 215:<br>ENTER THE NUMBER OF BIRTHS IN 2008 OR LATER.	NUMBER OF BIRTHS . . . . ☐<br>NONE 0	
224	Are you pregnant now?	YES . . . . 1<br>NO . . . . 2<br>UNSURE 8	2, 8 → 226
225	How many months pregnant are you?<br>RECORD NUMBER OF COMPLETED MONTHS.	MONTHS . . . . . . ☐☐	
226	CHECK 223:<br>ONE OR MORE BIRTHS IN 2008 OR LATER ☐<br>NO BIRTHS IN 2008 OR LATER ☐ → 426<br>Q. 223 IS BLANK ☐ → 426		426<br>426

SECTION 3. PREGNANCY AND INTERMITTENT PREVENTATIVE TREATMENT

NO.	QUESTIONS AND FILTERS	CODING CATEGORIES	SKIP
301	CHECK 215: ENTER IN THE TABLE THE NAME AND SURVIVAL STATUS OF THE MOST RECENT BIRTH.<br>Now I would like to ask some questions about your last pregnancy that resulted in a live birth.		
301A	FROM 212 AND 216 IN BIRTH HISTORY NUMBER 01:	MOST RECENT BIRTH<br>NAME ____________<br>LIVING ☐ DEAD ☐	
302	When you were pregnant with (NAME), did you see anyone for antenatal care for this pregnancy?	YES . . . . . . 1<br>NO 2	 → 303A
303	Whom did you see?<br>Anyone else?<br>PROBE TO IDENTIFY EACH TYPE OF PERSON AND RECORD ALL MENTIONED.	HEALTH PERSONNEL<br>DOCTOR . . . . . . A<br>NURSE/MIDWIFE . . . . . . B<br>MEDICAL ASSISTANT/ CLINICAL OFFICER . . . . . . C<br>NURSING AIDE . . . . . . D<br>OTHER PERSON<br>TRADITIONAL BIRTH ATTENDANT . . E<br>OTHER ____________ X<br>(SPECIFY)	→ 304
303A	What was the main reason why you did not see anyone for antenatal care?	CLINIC TOO FAR . . . . . . 1<br>HAD NO MONEY . . . . . . 2<br>HAD NO TIME . . . . . . 3<br>NOT AWARE HAD TO ATTEND . . . . . . 4<br>DID NOT WANT TO ATTEND . . . . . . 5<br>OTHER ____________ 6<br>(SPECIFY)<br>DON'T KNOW 8	
304	During this pregnancy, did you take any drugs to keep you from getting malaria?	YES . . . . . . 1<br>NO . . . . . . 2<br>DON'T KNOW 8	→ 305<br><br>→ 310
304A	What was the main reason why you did not did take any drugs to keep you from getting malaria during this pregnancy?	CLINIC TOO FAR . . . . . . 1<br>HAD NO MONEY . . . . . . 2<br>SIDE EFFECTS . . . . . . 3<br>NOT AWARE HAD TO TAKE ANY . . . 4<br>DID NOT WANT TO TAKE . . . . . . 5<br>OTHER ____________ 6<br>(SPECIFY)<br>DON'T KNOW . . . . . . 8<br>(ALL SKIP TO 310)	
305	What drugs did you take?<br>RECORD ALL MENTIONED.<br>IF TYPE OF DRUG IS NOT DETERMINED, SHOW TYPICAL ANTIMALARIAL DRUGS TO RESPONDENT.	SP/FANSIDAR . . . . . . A<br>CHLOROQUINE . . . . . . B<br>OTHER ____________ X<br>(SPECIFY)<br>DON'T KNOW . . . . . . Z	
306	CHECK 305:<br>SP/FANSIDAR TAKEN FOR MALARIA PREVENTION. CODE 'A' CIRCLED ☐ CODE 'A' NOT CIRCLED ☐		→ 310

307	How many times did you take (SP/Fansidar) during this pregnancy?	TIMES . . . . [ ][ ]	
307A	CHECK 307:<br>TOOK SP ONLY 1 TIME DURING THIS PREGNANCY<br>CODE '01' TIMES ENTERED [ ] ↓<br>OTHER [ ] → 308		308
307B	Why did you take (SP/Fansidar) only one time during this pregnancy?	CLINIC TOO FAR . . . . 1<br>HAD NO MONEY . . . . 2<br>SIDE EFFECTS . . . . 3<br>NOT AWARE HAD TO TAKE MORE . . . 4<br>DID NOT WANT TO TAKE . . . . 5<br>OTHER ______________ 6<br>(SPECIFY)<br>DON'T KNOW 8	
308	CHECK 303:<br>ANTENATAL CARE FROM HEALTH PERSONNEL DURING THIS PREGNANCY<br>CODE 'A', 'B', 'C', OR 'D' CIRCLED [ ] ↓<br>OTHER [ ] → 310		310
309	Did you get the (SP/Fansidar) during any antenatal care visit, during another visit to a health facility or from another source?	ANTENATAL VISIT . . . . 1<br>ANOTHER FACILITY VISIT . . . . 2<br>OTHER SOURCE 6	
310	CHECK 215 AND 216:<br>ONE OR MORE LIVING CHILDREN BORN IN 2008 OR LATER [ ] ↓ GO TO 401<br>NO LIVING CHILDREN BORN IN 2008 OR LATER [ ] → 426		426

SECTION 4. FEVER IN CHILDREN

401	CHECK 215: ENTER IN THE TABLE THE BIRTH HISTORY NUMBER, NAME, AND SURVIVAL STATUS OF EACH BIRTH IN 2008 OR LATER. ASK THE QUESTIONS ABOUT ALL OF THESE BIRTHS. BEGIN WITH THE MOST RECENT BIRTH. (IF THERE ARE MORE THAN 3 BIRTHS, USE AN ADDITIONAL QUESTIONNAIRE).<br>Now I would like to ask some questions about the health of your children born since January 2008. (We will talk about each separately.)			
402	BIRTH HISTORY NUMBER FROM 212 IN BIRTH HISTORY	MOST RECENT BIRTH<br>BIRTH HISTORY NUMBER [ ][ ]	SECOND MOST RECENT BIRTH<br>BIRTH HISTORY NUMBER [ ][ ]	THIRD MOST RECENT BIRTH<br>BIRTH HISTORY NUMBER [ ][ ]
403	FROM 212 AND 216	NAME ________________<br>LIVING [ ] DEAD [ ]<br>(GO TO 403 IN NEXT COLUMN OR, IF NO MORE BIRTHS, GO TO 426)	NAME ________________<br>LIVING [ ] DEAD [ ]<br>(GO TO 403 IN NEXT COLUMN OR, IF NO MORE BIRTHS, GO TO 426)	NAME ________________<br>LIVING [ ] DEAD [ ]<br>(GO TO 403 IN MOST RECENT COLUMN OF NEW QUESTIONNAIRE; OR, IF NO MORE BIRTHS, GO TO 426)
404	Has (NAME) been ill with a fever at any time in the last 2 weeks?	YES . . . . 1<br>NO . . . . 2<br>(GO TO 403 IN NEXT COLUMN OR, IF NO MORE BIRTHS, GO TO 426)<br>DON'T KNOW 8	YES . . . . 1<br>NO . . . . 2<br>(GO TO 403 IN NEXT COLUMN OR, IF NO MORE BIRTHS, GO TO 426)<br>DON'T KNOW 8	YES . . . . 1<br>NO . . . . 2<br>(GO TO 403 IN MOST RECENT COLUMN OF NEW QUESTIONNAIRE; OR, IF NO MORE BIRTHS, GO TO 426)<br>DON'T KNOW 8
405	At any time during the illness, did (NAME) have blood taken from his/her finger or heel for testing?	YES . . . . 1<br>NO . . . . 2<br>DON'T KNOW 8	YES . . . . 1<br>NO . . . . 2<br>DON'T KNOW 8	YES . . . . 1<br>NO . . . . 2<br>DON'T KNOW 8
406	Did you seek advice or treatment for the illness from any source?	YES . . . . 1<br>(SKIP TO 407)<br>NO 2	YES . . . . 1<br>(SKIP TO 407)<br>NO 2	YES . . . . 1<br>(SKIP TO 407)<br>NO 2
406A	Why have you not sought advice or treatment from any source?	CHILD JUST FELL ILL . . A<br>CHILD NOT VERY ILL . . B<br>CLINIC TOO FAR . . . . C<br>HAVE NO MONEY . . . D<br>WAITING FOR CHILD'S FATHER . . . . E<br>DON'T KNOW WHAT TO DO . . . . F<br>ALREADY HAD MEDICINE AT HOME . G<br>OTHER _____________ X<br>(SPECIFY)<br>SKIP TO 410	CHILD JUST FELL ILL . . A<br>CHILD NOT VERY ILL . . B<br>CLINIC TOO FAR . . . . C<br>HAVE NO MONEY . . . D<br>WAITING FOR CHILD'S FATHER . . . . E<br>DON'T KNOW WHAT TO DO . . . . F<br>ALREADY HAD MEDICINE AT HOME . G<br>OTHER ___________ X<br>(SPECIFY)<br>SKIP TO 410	CHILD JUST FELL ILL . . A<br>CHILD NOT VERY ILL . . B<br>CLINIC TOO FAR . . . . C<br>HAVE NO MONEY . . . D<br>WAITING FOR CHILD'S FATHER . . . . E<br>DON'T KNOW WHAT TO DO . . . . F<br>ALREADY HAD MEDICINE AT HOME . G<br>OTHER ___________ X<br>(SPECIFY)<br>SKIP TO 410

NO.	QUESTIONS AND FILTERS	MOST RECENT BIRTH<br>NAME ________	SECOND MOST RECENT BIRTH<br>NAME ________	THIRD MOST RECENT BIRTH<br>NAME ________
407	Where did you seek advice or treatment?<br><br>Anywhere else?<br><br>PROBE TO IDENTIFY EACH TYPE OF SOURCE.<br><br>IF UNABLE TO DETERMINE IF PUBLIC OR PRIVATE SECTOR, WRITE THE NAME OF THE PLACE.<br><br>________<br>(NAME OF PLACE(S))	PUBLIC SECTOR (GOVERNMENT)<br>GOVT HOSPITAL . . A<br>GOVT HEALTH CENTER . . . . . . B<br>GOVT HEALTH POST . . . . C<br>MOBILE CLINIC . . . D<br>COMMUNITY HEALTH WORKER(VHT) . E<br>OTHER PUBLIC SECTOR ________ F<br>(SPECIFY)<br>PUBLIC SECTOR (PNFP)<br>HOSPITAL . . . . . . G<br>HEALTH CENTER . H<br>PRIVATE MEDICAL SECTOR<br>PVT HOSPITAL/ CLINIC . . . . I<br>PHARMACY . . . J<br>MOBILE CLINIC . . . K<br>FIELDWORKER . . L<br>OTHER PRIVATE MED. SECTOR ________ M<br>(SPECIFY)<br>OTHER SOURCE<br>SHOP . . . . N<br>TRADITIONAL PRACTITIONER . O<br>MARKET . . . . . . P<br>OTHER ________ X<br>(SPECIFY)	PUBLIC SECTOR (GOVERNMENT)<br>GOVT HOSPITAL . . A<br>GOVT HEALTH CENTER . . . . . . B<br>GOVT HEALTH POST . . . . C<br>MOBILE CLINIC . . . D<br>COMMUNITY HEALTH WORKER(VHT) . E<br>OTHER PUBLIC SECTOR ________ F<br>(SPECIFY)<br>PUBLIC SECTOR (PNFP)<br>HOSPITAL . . . . . . G<br>HEALTH CENTER . H<br>PRIVATE MEDICAL SECTOR<br>PVT HOSPITAL/ CLINIC . . . . I<br>PHARMACY . . . J<br>MOBILE CLINIC . . . K<br>FIELDWORKER . . L<br>OTHER PRIVATE MED. SECTOR ________ M<br>(SPECIFY)<br>OTHER SOURCE<br>SHOP . . . . N<br>TRADITIONAL PRACTITIONER . O<br>MARKET . . . . . . P<br>OTHER ________ X<br>(SPECIFY)	PUBLIC SECTOR (GOVERNMENT)<br>GOVT HOSPITAL . . A<br>GOVT HEALTH CENTER . . . . . . B<br>GOVT HEALTH POST . . . . C<br>MOBILE CLINIC . . . D<br>COMMUNITY HEALTH WORKER(VHT) . E<br>OTHER PUBLIC SECTOR ________ F<br>(SPECIFY)<br>PUBLIC SECTOR (PNFP)<br>HOSPITAL . . . . . . G<br>HEALTH CENTER . H<br>PRIVATE MEDICAL SECTOR<br>PVT HOSPITAL/ CLINIC . . . . I<br>PHARMACY . . . J<br>MOBILE CLINIC . . . K<br>FIELDWORKER . . L<br>OTHER PRIVATE MED. SECTOR ________ M<br>(SPECIFY)<br>OTHER SOURCE<br>SHOP . . . . N<br>TRADITIONAL PRACTITIONER . O<br>MARKET . . . . . . P<br>OTHER ________ X<br>(SPECIFY)
408	CHECK 407:	TWO OR MORE CODES CIRCLED ☐<br>ONLY ONE CODE CIRCLED ☐ (SKIP TO 410)	TWO OR MORE CODES CIRCLED ☐<br>ONLY ONE CODE CIRCLED ☐ (SKIP TO 410)	TWO OR MORE CODES CIRCLED ☐<br>ONLY ONE CODE CIRCLED ☐ (SKIP TO 410)
409	Where did you first seek advice or treatment?<br><br>USE LETTER CODE FROM 407.	FIRST PLACE . . . ☐	FIRST PLACE . . . ☐	FIRST PLACE . . . ☐
410	At any time during the illness, did (NAME) take any drugs for the illness?	YES . . . . 1<br>NO . . . . 2<br>(GO BACK TO 403 IN NEXT COLUMN; OR, IF NO MORE BIRTHS, GO TO 426)<br>DON'T KNOW 8	YES . . . . 1<br>NO . . . . 2<br>(GO BACK TO 403 IN NEXT COLUMN; OR, IF NO MORE BIRTHS, GO TO 426)<br>DON'T KNOW 8	YES . . . . 1<br>NO . . . . 2<br>(GO TO 403 IN MOST RECENT COLUMN OF NEW QUESTIONNAIRE; OR, IF NO MORE BIRTHS, GO TO 426)<br>DON'T KNOW 8

NO.	QUESTIONS AND FILTERS	MOST RECENT BIRTH<br>NAME ________	SECOND MOST RECENT BIRTH<br>NAME ________	THIRD MOST RECENT BIRTH<br>NAME ________

NO.	QUESTIONS AND FILTERS	MOST RECENT BIRTH<br>NAME ________	SECOND MOST RECENT BIRTH<br>NAME ________	THIRD MOST RECENT BIRTH<br>NAME ________

411	What drugs did (NAME) take?<br><br>Any other drugs?<br><br>RECORD ALL MENTIONED.	ANTIMALARIAL DRUGS<br>SP/FANSIDAR . . . A<br>CHLOROQUINE . . B<br>AMODIAQUINE . . C<br>QUININE . . . . D<br>COARTEM/ACT . . E<br>OTHER ANTI-MALARIAL<br>________ . . . F<br>(SPECIFY)<br>ANTIBIOTIC DRUGS<br>PILL/SYRUP . . . G<br>INJECTION . . . . . . H<br>OTHER DRUGS<br>ASPIRIN . . . . I<br>PANADOL . . . . . . J<br>IBUPROFEN . . . . . . K<br>OTHER ________ X<br>(SPECIFY)<br>DON'T KNOW . . . . . . Z	ANTIMALARIAL DRUGS<br>SP/FANSIDAR . . . A<br>CHLOROQUINE . . B<br>AMODIAQUINE . . C<br>QUININE . . . . D<br>COARTEM/ACT . . E<br>OTHER ANTI-MALARIAL<br>________ . . . F<br>(SPECIFY)<br>ANTIBIOTIC DRUGS<br>PILL/SYRUP . . . G<br>INJECTION . . . . . . H<br>OTHER DRUGS<br>ASPIRIN . . . . I<br>PANADOL . . . . . . J<br>IBUPROFEN . . . K<br>OTHER ________ X<br>(SPECIFY)<br>DON'T KNOW . . . . . . Z	ANTIMALARIAL DRUGS<br>SP/FANSIDAR . . . A<br>CHLOROQUINE . . B<br>AMODIAQUINE . . C<br>QUININE . . . . D<br>COARTEM/ACT . . E<br>OTHER ANTI-MALARIAL<br>________ . . . F<br>(SPECIFY)<br>ANTIBIOTIC DRUGS<br>PILL/SYRUP . . . G<br>INJECTION . . . . . . H<br>OTHER DRUGS<br>ASPIRIN . . . . I<br>PANADOL . . . . . . J<br>IBUPROFEN . . . . . . K<br>OTHER ________ X<br>(SPECIFY)<br>DON'T KNOW . . . . . . Z
412	CHECK 411:<br>ANY CODE A-F CIRCLED?	YES ☐ ↓<br>NO ☐ (GO BACK TO 403 IN NEXT COLUMN; OR, IF NO MORE BIRTHS, GO TO 426)	YES ☐ ↓<br>NO ☐ (GO BACK TO 403 IN NEXT COLUMN; OR, IF NO MORE BIRTHS, GO TO 426)	YES ☐ ↓<br>NO ☐ (GO TO 403 IN MOST RECENT COLUMN OF NEW QUESTIONNAIRE; OR, IF NO MORE BIRTHS, GO TO 426)
413	CHECK 411:<br><br>SP/FANSIDAR ('A') GIVEN	CODE 'A' CIRCLED ☐ ↓<br>CODE 'A' NOT CIRCLED ☐ (SKIP TO 415)	CODE 'A' CIRCLED ☐ ↓<br>CODE 'A' NOT CIRCLED ☐ (SKIP TO 415)	CODE 'A' CIRCLED ☐ ↓<br>CODE 'A' NOT CIRCLED ☐ (SKIP TO 415)
414	How long after the fever started did (NAME) first take (SP/Fansidar)?	SAME DAY . . . . 0<br>NEXT DAY . . . . 1<br>TWO DAYS AFTER FEVER . . . . 2<br>THREE OR MORE DAYS AFTER FEVER . . . . 3<br>DON'T KNOW . . . 8	SAME DAY . . . . 0<br>NEXT DAY . . . . 1<br>TWO DAYS AFTER FEVER . . . . 2<br>THREE OR MORE DAYS AFTER FEVER . . . . 3<br>DON'T KNOW . . . 8	SAME DAY . . . . 0<br>NEXT DAY . . . . 1<br>TWO DAYS AFTER FEVER . . . . 2<br>THREE OR MORE DAYS AFTER FEVER . . . . 3<br>DON'T KNOW . . . 8
415	CHECK 411:<br><br>CHLOROQUINE ('B') GIVEN	CODE 'B' CIRCLED ☐ ↓<br>CODE 'B' NOT CIRCLED ☐ (SKIP TO 417)	CODE 'B' CIRCLED ☐ ↓<br>CODE 'B' NOT CIRCLED ☐ (SKIP TO 417)	CODE 'B' CIRCLED ☐ ↓<br>CODE 'B' NOT CIRCLED ☐ (SKIP TO 417)

NO.	QUESTIONS AND FILTERS	MOST RECENT BIRTH<br>NAME ________________	SECOND MOST RECENT BIRTH<br>NAME ________________	THIRD MOST RECENT BIRTH<br>NAME ________________
NO.	QUESTIONS AND FILTERS	MOST RECENT BIRTH<br>NAME ________________	SECOND MOST RECENT BIRTH<br>NAME ________________	THIRD MOST RECENT BIRTH<br>NAME ________________
416	How long after the fever started did (NAME) first take chloroquine?	SAME DAY . . . . 0<br>NEXT DAY . . . . 1<br>TWO DAYS AFTER FEVER . . . . 2<br>THREE OR MORE DAYS AFTER FEVER . . . . 3<br>DON'T KNOW . . . 8	SAME DAY . . . . 0<br>NEXT DAY . . . . 1<br>TWO DAYS AFTER FEVER . . . . 2<br>THREE OR MORE DAYS AFTER FEVER . . . . 3<br>DON'T KNOW . . . 8	SAME DAY . . . . 0<br>NEXT DAY . . . . 1<br>TWO DAYS AFTER FEVER . . . . 2<br>THREE OR MORE DAYS AFTER FEVER . . . . 3<br>DON'T KNOW . . . 8
417	CHECK 411:<br><br>AMODIAQUINE ('C') GIVEN	CODE 'C' CIRCLED ☐ ↓<br>CODE 'C' NOT CIRCLED ☐ (SKIP TO 419)	CODE 'C' CIRCLED ☐ ↓<br>CODE 'C' NOT CIRCLED ☐ (SKIP TO 419)	CODE 'C' CIRCLED ☐ ↓<br>CODE 'C' NOT CIRCLED ☐ (SKIP TO 419)
418	How long after the fever started did (NAME) first take amodiaquine?	SAME DAY . . . . 0<br>NEXT DAY . . . . 1<br>TWO DAYS AFTER FEVER . . . . 2<br>THREE OR MORE DAYS AFTER FEVER . . . . 3<br>DON'T KNOW . . . 8	SAME DAY . . . . 0<br>NEXT DAY . . . . 1<br>TWO DAYS AFTER FEVER . . . . 2<br>THREE OR MORE DAYS AFTER FEVER . . . . 3<br>DON'T KNOW . . . 8	SAME DAY . . . . 0<br>NEXT DAY . . . . 1<br>TWO DAYS AFTER FEVER . . . . 2<br>THREE OR MORE DAYS AFTER FEVER . . . . 3<br>DON'T KNOW . . . 8
419	CHECK 411:<br><br>QUININE ('D') GIVEN	CODE 'D' CIRCLED ☐ ↓<br>CODE 'D' NOT CIRCLED ☐ (SKIP TO 421)	CODE 'D' CIRCLED ☐ ↓<br>CODE 'D' NOT CIRCLED ☐ (SKIP TO 421)	CODE 'D' CIRCLED ☐ ↓<br>CODE 'D' NOT CIRCLED ☐ (SKIP TO 421)
420	How long after the fever started did (NAME) first take quinine?	SAME DAY . . . . 0<br>NEXT DAY . . . . 1<br>TWO DAYS AFTER FEVER . . . . 2<br>THREE OR MORE DAYS AFTER FEVER . . . . 3<br>DON'T KNOW . . . 8	SAME DAY . . . . 0<br>NEXT DAY . . . . 1<br>TWO DAYS AFTER FEVER . . . . 2<br>THREE OR MORE DAYS AFTER FEVER . . . . 3<br>DON'T KNOW . . . 8	SAME DAY . . . . 0<br>NEXT DAY . . . . 1<br>TWO DAYS AFTER FEVER . . . . 2<br>THREE OR MORE DAYS AFTER FEVER . . . . 3<br>DON'T KNOW . . . 8
421	CHECK 411:<br><br>COMBINATION WITH COARTEM/ACT ('E') GIVEN	CODE 'E' CIRCLED ☐ ↓<br>CODE 'E' NOT CIRCLED ☐ (SKIP TO 423)	CODE 'E' CIRCLED ☐ ↓<br>CODE 'E' NOT CIRCLED ☐ (SKIP TO 423)	CODE 'E' CIRCLED ☐ ↓<br>CODE 'E' NOT CIRCLED ☐ (SKIP TO 423)
422	How long after the fever started did (NAME) first take COARTEM/ACT?	SAME DAY . . . . 0<br>NEXT DAY . . . . 1<br>TWO DAYS AFTER FEVER . . . . 2<br>THREE OR MORE DAYS AFTER FEVER . . . . 3<br>DON'T KNOW . . . 8	SAME DAY . . . . 0<br>NEXT DAY . . . . 1<br>TWO DAYS AFTER FEVER . . . . 2<br>THREE OR MORE DAYS AFTER FEVER . . . . 3<br>DON'T KNOW . . . 8	SAME DAY . . . . 0<br>NEXT DAY . . . . 1<br>TWO DAYS AFTER FEVER . . . . 2<br>THREE OR MORE DAYS AFTER FEVER . . . . 3<br>DON'T KNOW . . . 8

NO.	QUESTIONS AND FILTERS	MOST RECENT BIRTH<br>NAME ________________	SECOND MOST RECENT BIRTH<br>NAME ________________	THIRD MOST RECENT BIRTH<br>NAME ________________
NO.	QUESTIONS AND FILTERS	MOST RECENT BIRTH<br>NAME ________________	SECOND MOST RECENT BIRTH<br>NAME ________________	THIRD MOST RECENT BIRTH<br>NAME ________________
422A	For how many day did (NAME) take COATREM/ACT?	DAYS . . . [ ][ ]<br><br>STILL TAKING . . . . . . 95<br>DON'T KNOW 98	DAYS . . . [ ][ ]<br><br>STILL TAKING . . . . . . 95<br>DON'T KNOW 98	DAYS . . . [ ][ ]<br><br>STILL TAKING . . . . . . 95<br>DON'T KNOW 98
423	CHECK 411:<br><br>OTHER ANTIMALARIAL ('F') GIVEN	CODE 'F' CIRCLED [ ]<br>CODE 'F' NOT CIRCLED [ ] (GO BACK TO 403 IN NEXT COLUMN; OR, IF NO MORE BIRTHS, GO TO 426)	CODE 'F' CIRCLED [ ]<br>CODE 'F' NOT CIRCLED [ ] (GO BACK TO 403 IN NEXT COLUMN; OR, IF NO MORE BIRTHS, GO TO 426)	CODE 'F' CIRCLED [ ]<br>CODE 'F' NOT CIRCLED [ ] (GO TO 403 IN MOST RECENT COLUMN OF NEW QUESTIONNAIRE; OR, IF NO MORE BIRTHS, GO TO 426)
424	How long after the fever started did (NAME) first take (OTHER ANTIMALARIAL)?	SAME DAY . . . . 0<br>NEXT DAY . . . . 1<br>TWO DAYS AFTER FEVER . . . . 2<br>THREE OR MORE DAYS AFTER FEVER . . . . 3<br>DON'T KNOW . . . 8	SAME DAY . . . . 0<br>NEXT DAY . . . . 1<br>TWO DAYS AFTER FEVER . . . . 2<br>THREE OR MORE DAYS AFTER FEVER . . . . 3<br>DON'T KNOW . . . 8	SAME DAY . . . . 0<br>NEXT DAY . . . . 1<br>TWO DAYS AFTER FEVER . . . . 2<br>THREE OR MORE DAYS AFTER FEVER . . . . 3<br>DON'T KNOW . . . 8
425		GO BACK TO 403 IN NEXT COLUMN; OR, IF NO MORE BIRTHS, GO TO 426.	GO BACK TO 403 IN NEXT COLUMN; OR, IF NO MORE BIRTHS, GO TO 426.	GO TO 403 IN MOST RECENT COLUMN OF NEW QUESTIONNAIRE; OR, IF NO MORE BIRTHS, GO TO 426.

NO.	QUESTIONS AND FILTERS	CODING CATEGORIES	SKIP
426	I would like to ask you a few questions about fever in children.<br><br>When a child is sick with fever, how long after the fever begins should the child be taken for treatment?	SAME DAY . . . . 01<br>NEXT DAY . . . . 02<br>TWO DAYS AFTER ONSET<br>OF FEVER . . . . 03<br>THREE OR MORE DAYS AFTER<br>ONSET OF FEVER . . . . 04<br>FEVER IS NORMAL IN CHILDREN,<br>NO TREATMENT NECESSARY . . . 05<br>DEPENDS ON HOW SERIOUS THE<br>FEVER IS . . . . 06<br>OTHER ______________ 96<br>(SPECIFY)<br>DON'T KNOW 98	
427	In your opinion, what causes malaria?<br><br>PROBE: Anything else?<br><br>RECORD ALL MENTIONED	MOSQUITO BITES . . . . A<br>PARASITE . . . . B<br>EATING MAIZE . . . . C<br>EATING MANGOES . . . . D<br>EATING DIRTY FOOD . . . . E<br>DRINKING UNBOILED WATER . . . . F<br>GETTING SOAKED WITH RAIN . . . . . G<br>COLD/CHANGING WEATHER . . . . H<br>WITCHCRAFT . . . . I<br>CONTACT WITH INFECTED PERSON . . J<br>GERM . . . . K<br>OTHER ______________ X<br>(SPECIFY)<br>DON'T KNOW . . . . Z	
428	Are there ways to avoid getting malaria?	YES . . . . 1<br>NO 2	 → 431
429	What are the ways to avoid getting malaria?<br><br>PROBE: Anything else?<br><br>RECORD ALL MENTIONED	SLEEP UNDER MOSQUITO NET . . . . . A<br>SLEEP UNDER AN INSECTICIDE<br>TREATED NET . . . . B<br>TAKING PREVENTIVE<br>MEDICATION . . . . C<br>USE MOSQUITO REPELLANT . . . . D<br>SPRAYING HOUSE WITH<br>INSECTICIDE . . . . E<br>USING MOSQUITO COILS . . . . F<br>DESTROY MOSQUITO<br>BREEDING SITES . . . . G<br>OTHER ______________ X<br>(SPECIFY)<br>DON'T KNOW . . . . Z	
430	What medicine may be given to a pregnant woman to help her avoid getting malaria?<br><br>RECORD ALL MENTIONED	SP/FANSIDAR . . . . A<br>CHLOROQUINE . . . . B<br>CHLOROQUINE<br>W/ FANSIDAR . . . . C<br>COARTEM/ACT . . . . D<br>OTHER ______________ X<br>(SPECIFY)<br>DON'T KNOW . . . . Z	
431	CHECK 430 SP/FANSIDAR MENTIONED<br>CODE 'A' CIRCLED ☐ ↓<br>CODE 'A' NOT CIRCLED ☐ →		433
432	How many times does a woman need to take SP/FANSIDAR during her pregnancy to avoid getting malaria?	NUMBER OF<br>TIMES . . . . [ ][ ]<br>DON'T KNOW 98	
433	RECORD THE TIME.	HOUR . . . . [ ][ ]<br>MINUTES . . . . [ ][ ]	

INTERVIEWER'S OBSERVATIONS

TO BE FILLED IN AFTER COMPLETING INTERVIEW

COMMENTS ABOUT RESPONDENT:

COMMENTS ON SPECIFIC QUESTIONS:

ANY OTHER COMMENTS:

SUPERVISOR'S OBSERVATIONS

NAME OF SUPERVISOR: ____________________ DATE: ____________________
